# DrugPred_RNA—A Tool for Structure-Based Druggability Predictions for RNA Binding Sites

**DOI:** 10.1021/acs.jcim.1c00155

**Published:** 2021-07-21

**Authors:** Illimar Hugo Rekand, Ruth Brenk

**Affiliations:** Department of Biomedicine, University of Bergen, Jonas Lies Vei, 5020 Bergen, Norway

## Abstract

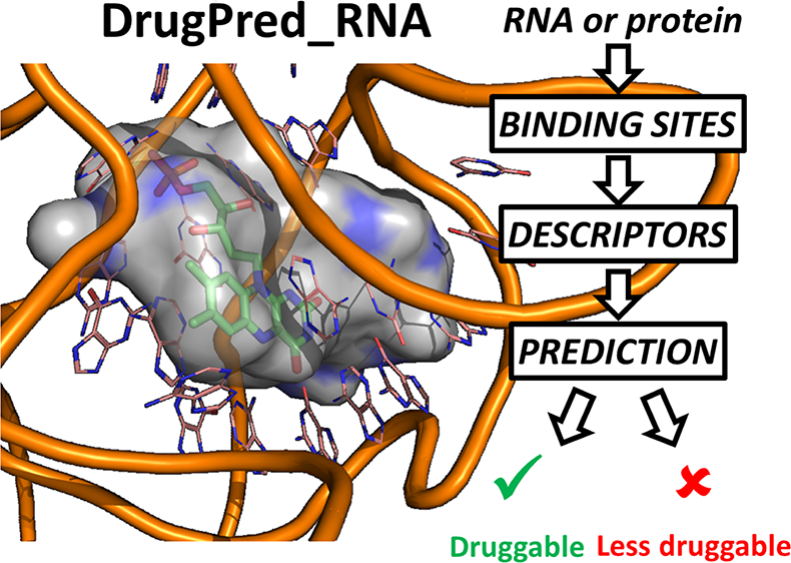

RNA is an emerging target for drug discovery. However, like for proteins, not all RNA binding sites are equally suited to be addressed with conventional drug-like ligands. To this end, we have developed the structure-based druggability predictor DrugPred_RNA to identify druggable RNA binding sites. Due to the paucity of annotated RNA binding sites, the predictor was trained on protein pockets, albeit using only descriptors that can be calculated for both RNA and protein binding sites. DrugPred_RNA performed well in discriminating druggable from less druggable binding sites for the protein set and delivered predictions for selected RNA binding sites that agreed with manual assignment. In addition, most drug-like ligands contained in an RNA test set were found in pockets predicted to be druggable, further adding confidence to the performance of DrugPred_RNA. The method is robust against conformational and sequence changes in the binding sites and can contribute to direct drug discovery efforts for RNA targets.

## Introduction

**Figure 1:**
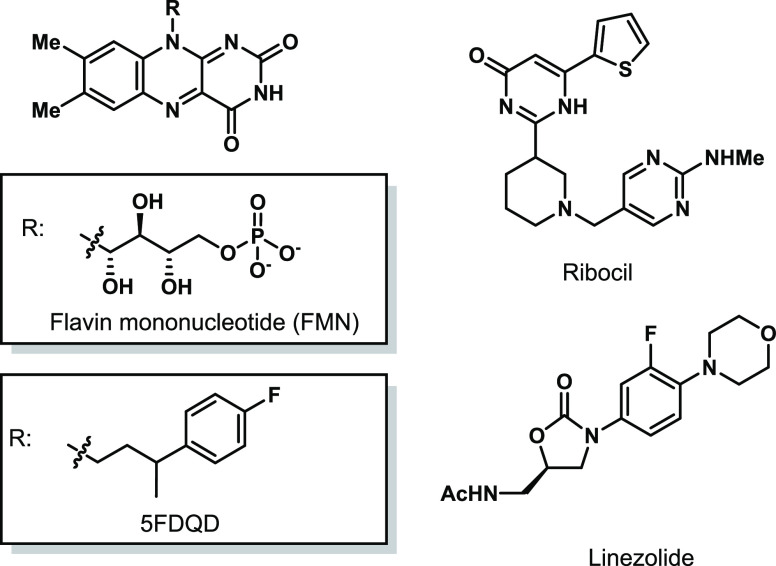
Examples of RNA-binding small molecules. FMN is the natural ligand for the FMN riboswitch,^15^ while 5FDQD and ribocil are synthetic ligands for the same target.^13,14^ Linezolide, an FDA-approved antibiotic, targets bacterial ribosomal RNA, thus inhibiting protein synthesis.^8^

The vast majority of targets for approved drugs are proteins.^1,2^ However, in recent years, it has been increasingly realized that RNAs also constitute promising drug targets as they play a key role in many biological processes, can fold into diverse 3D structures, and specifically recognize small molecules.^3−6^ By targeting RNA, the functions of currently undruggable protein-mediated pathways and the noncoding transcriptome can be modulated, and thus, the size of the druggable genome can be increased considerably.^3^ A prime example of an RNA drug target is the bacterial ribosome, where protein synthesis is inhibited through binding of small molecules.^7^ This is illustrated by linezolid, an FDA-approved antibiotic, which acts by binding to ribosomal RNA (Figure 1).^8^ Another active research area is the discovery of RNA-binding splicing modifiers for the treatment of spinal muscular atrophy with several compounds in clinical trials.^9,10^ Riboswitches, which are noncoding RNA structures in the 5′ untranslated region and regulate gene expression through metabolite binding, are new RNA drug targets for antibiotics.^11,12^ For example, compounds binding to the flavin mononucleotide (FMN) riboswitch, e.g., ribocil and 5FDQD, have been shown to kill bacteria (Figure 1).^13,14^ Riboflavin is known to bind to both the FMN riboswitch and riboflavin kinase. In both binding sites, the ligand is recognized in a similar way, forming hydrophobic contacts and hydrogen bonds between the surrounding residues and the pteridine ring system, the dimethylbenzene ring, and the ribose chain. This fact nicely illustrates the capability of RNA to make specific molecular interactions with a wide variety of functional groups and ligand surfaces.^3^

When targeting RNA, the question arises as to which targets are best suited for drug discovery and where in chemical space to look for potent ligands. Analysis of RNA-binding small molecules has revealed that some RNA ligands have drug-like properties comparable to FDA-approved drugs, while others lie outside this space.^4,16^ Warner *et al.* have argued that RNA targets that bind such drug-like molecules and are thus deemed to be “ligandable” hold the greatest promise.^3^ Consequently, tools are needed to identify such targets.

Targets are commonly considered to be “ligandable” or “druggable” if they possess binding sites that allow them to bind orally bioavailable drugs with high affinity.^17,18^ The terms to name such pockets are hotly debated, and several alternative terms such as “bindability”, “tractability”, or “chemical tractability” have been proposed.^18^ We will use the term “druggability” throughout this manuscript because it is the prevalent term used in the literature. Druggability is not an absolute property, and for other pockets, potent drugs can be developed, albeit larger efforts might be required. Accordingly, we will label pockets that are not classified to be druggable as “less druggable”.

Over the last few years, several methods have been reported that are able to segregate druggable pockets from less druggable ones based on the 3D structure of the binding site.^18^ Typically, these methods use descriptors describing the hydrophobicity, size, and shape of the pockets to classify them using machine learning methods. As training and validation sets, protein pockets that have been assigned to either category are used. One of these methods, the DLID (drug-like density) measure,^19^ has also been applied to analyze RNA pockets. DLID uses PocketFinder^20^ to identify potential binding sites and the descriptors volume, buriedness, and hydrophobicity to estimate how likely a pocket is to bind a drug-like molecule. Warner *et al.* used this approach to illustrate the diversity of selected RNA binding sites.^3^ Hewitt *et al.* conducted a comprehensive analysis of RNA structures in the PDB using the same method and concluded that many RNAs contain pockets that are likely suitable for small molecule binding.^21^ However, they did not distinguish between the binding of drug-like ligands and other molecules.

In our group, we have developed DrugPred as a structure-based druggability prediction method for protein binding sites.^22,23^ DrugPred describes the size and shape of the binding site using a “superligand” as a negative print, which is obtained by merging predicted binding modes of drug molecules that were docked into the pocket using only steric constraints. Descriptors encoding the polarity and size of the pocket are subsequently calculated based on the superligand and used to predict the druggability of the binding site. DrugPred was trained and validated on a set of nonredundant druggable and less druggable protein binding sites (NRDLD), which has become a standard in the field. In comparison studies, DrugPred performed at least equally well than other methods and achieved an accuracy of about 90%.^22,24,25^

A hurdle when developing a druggability predictor for RNA is the paucity of training and validation data. Compared to the protein field, there are very little data about ligands binding to RNA and even less data that can be accessed in an efficient way. In the Protein Data Bank (PDB),^26^ only 43 crystal structures containing only RNA as macromolecule are annotated with affinity data from PDBbind^27^ mapping to about 20 unique sequences. The NALDB and SMMRNA databases contain affinities of small molecules binding to RNA extracted from the literature.^28,29^ However, it is not possible to download the data for further processing. The R-BIND database links binding data to RNA crystal structures, but for only five of the ligands in this database is a complex structure available in the PDB.^30^ As the principles of molecular recognition are universal, the lack of RNA data can potentially be overcome by training a predictor on protein binding sites as long as only descriptors that can be calculated for both types of pockets are used.

Here, we adopted DrugPred for druggability predictions of RNA binding sites. As some of the original DrugPred descriptors could only be calculated for amino acids (the hydrophobicity indices of amino acids and the relative occurrence of hydrophobic amino acids in the pockets),^22^ we have implemented alternative descriptors and thus made a prediction software that is applicable to both protein and RNA binding sites. Due to the paucity of suitable RNA data, we opted to train our modified DrugPred model, which we termed DrugPred_RNA, on our previously derived NRDLD protein binding site set. For machine learning, the decision tree algorithm XGBoost (eXtreme Gradient Boosting) was used.^31^ In the absence of a benchmarking set for RNA druggability predictions, we compiled a set containing RNA and ribosome binding sites from the PDB for validating the performance of DrugPred_RNA on RNA pockets. Here, we present the construction of DrugPred_RNA, the compilation of RNA sets for druggability predictions, and the validation results with the protein and RNA sets. Further, we discuss the implications of this study for RNA-targeted drug discovery.

## Methods

Scripts to download crystal structures from the PDB, process them, and calculate ligand and binding site descriptors were written using Python 3.6.8 with the Biopython (1.73) and RDKit (2019.09.1) libraries.^32,33^

### NRDLD Set for Training and Validation

Our NRDLD set with the most recent modifications was used to train and test a druggability predictor on protein targets.^22,23^ In brief, this set contains 110 small molecule binding sites. The proteins in the set have a maximum sequence similarity of 60% to each other, and 68 of the binding sites were previously annotated to be druggable and 42 to be less druggable based on data mining and available literature. This set was split into a training set containing 75 pockets (47 druggable/28 less druggable) and a test set containing 35 pockets (21 druggable/14 less druggable) as done before. The binding sites and surrounding residues were carved out of the CIF files downloaded from the PDB by keeping all residues with an atom within 15 Å of the ligand to reduce the file size. The isolated parts of the structures together with co-factors and metal ions if present were saved in the PDB format and used for generating the superligand and calculating descriptors as described below.

### Superligand Generation

A superligand as a negative print of the binding site was obtained as done previously with minor modifications.^22^ In brief, a set of approved drug molecules was docked into the pocket that contained the bound ligand in the original crystal structure using DOCK 3.6.^34^ Since the aim of docking was solely to obtain information about the shape and the volume of the binding site, all receptor atoms were set to carbon atoms and assigned a partial charge of 0. Subsequently, compounds for which a docking pose was obtained and for which the ratio of van der Waals (VDW) score to number of heavy atoms was ≤−1.3 were merged into a superligand. This cutoff was chosen to ensure that only ligands that filled the pocket were kept. To minimize the number of atoms in the final superligand, during the merging process, only atoms adhering to all of the following criteria were retained: (1) the atom had to be a nonhydrogen atom, (2) at least two atoms coming from different docked compounds had to be closer than 1.2 Å, and (3) only one of the atoms within 1.2 Å from other atoms was kept. If no docked ligands passed these filters, the ligand contained in the original complex structure was used as the superligand. This was the case in 125 instances in the RNA data set and 342 instances in the ribosomal data set.

### Descriptor Calculation

The binding site and buried superligand atoms were determined based on the superligand. For that purpose, using FreeSASA^35^ as implemented in RDKit, the solvent accessible surface area (SASA) of each receptor and superligand atom in the superligand-bound and -unbound state was calculated using a 1.0 Å probe radius and ProtOr radii.^36^ All receptor atoms for which the SASA differed between superligand-bound and -unbound state were assigned as being binding site atoms. Further, the SASA of all superligand atoms in the free state was calculated. Superligand atoms with a SASA >0 were assigned as surface atoms, and those with a SASA = 0 were assigned as buried superligand atoms.

Using superligand and binding site atoms as input, descriptors describing the size, shape, and polarity of the pocket were calculated (Table S1). For shape descriptors that are not based on the surface area or the number of receptor or superligand atoms, the Descriptors3D module of RDKit was used. For calculating polarity descriptors, we considered all carbon, phosphor, and sulfur atoms in addition to nitrogen atoms of the RNA bases that are bound to the ribose to be hydrophobic and all oxygen atoms of amino acids, ribose sugars, and phosphate groups in addition to nonaromatic nitrogen atoms of amino acids to be polar. The SASA values of these atoms were calculated with FreeSASA using the same settings as described above. The side chains of histidine and tryptophane residues as well as the RNA bases are known to form hydrogen bonds in the plane of the heterocycles, while parallel to this plane, they engage in pi-stacking interactions that are more hydrophobic in nature. To account for this ambivalent behavior, the SASA of endocyclic aromatic nitrogen atoms of the bases and amino acid side chains and exocyclic oxygen and nitrogen atoms of the bases was split into a hydrophobic and a polar contribution in the following way. The SASA of these atoms was calculated in both the absence (SASA_total) and the presence (SASA_pol) of two blocking carbon atoms that were placed perpendicular to the plane of the aromatic ring with a 1.70 Å distance from the atom of interest. The area SASA_pol was considered to belong to a polar atom, while the difference SASA_total – SASA_pol was considered to belong to a hydrophobic atom. Similarly, if more than half of the SASA of an atom was deemed to be hydrophobic, the atom was included in the hydrophobic binding site atom count.

### Training the Predictive Model Using Decision Trees

Machine learning was carried out using the XGBoost^31^ package in R,^37^ a scalable machine learning system for tree boosting. In brief, the method is based on initially creating multiple decision trees that are evolved over time into a model with increased predictive power. As a learning objective, logistic regression for binary classification with output probability was used. Thus, all binding sites obtained a score between 0.0 and 1.0, whereas pockets with a score ≥ 0.5 were labeled druggable and pockets with a score < 0.5 were labeled as less druggable. Divergent from the default settings, the following parameters were used for training the model:

- *Max_depth* = 3 (maximum depth of trees)
- *Scale_pos_weight* = 0.63 (adjusts for the skewness between druggable and less druggable binding sites in the training set)
- *Early_stopping_rounds* = 20 (Validation metric needs to improve at least once in every **20** rounds to continue training.)

**Figure 2:**
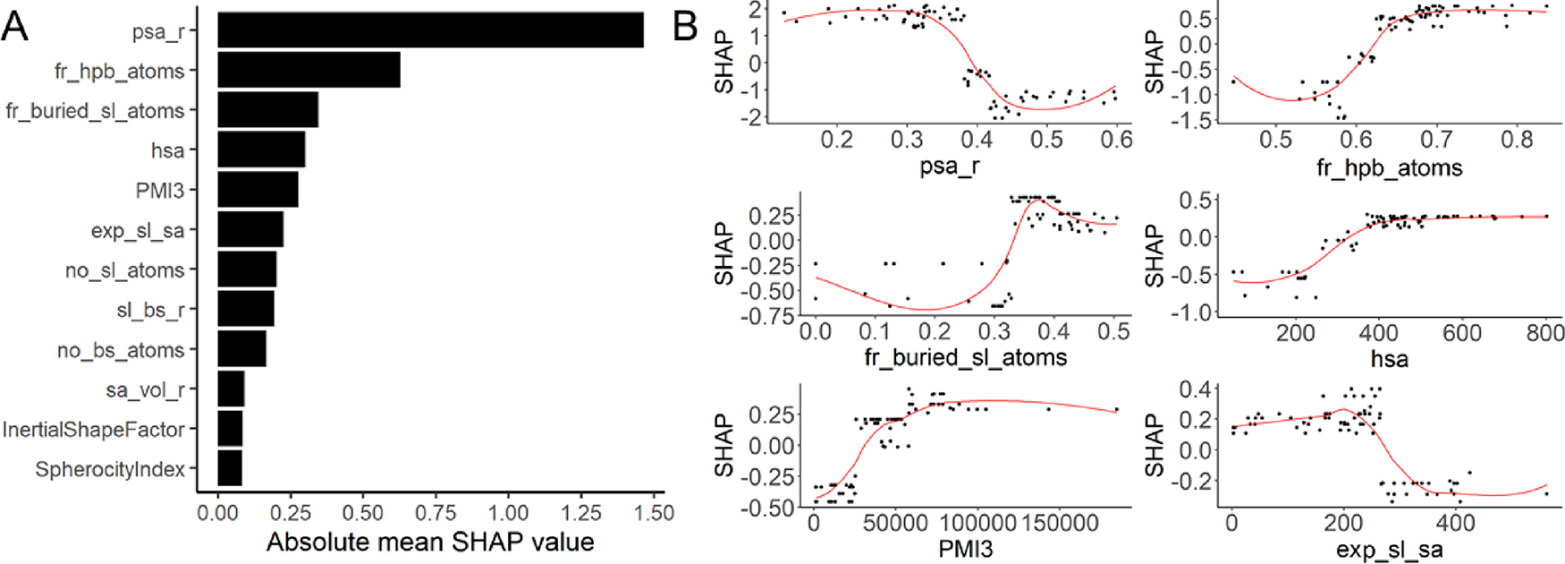
SHAP values for the DrugPred_RNA model. (A) Absolute mean SHAP values for each descriptor ranked from the highest to lowest impact on the model output. (B) Individual SHAP values for each pocket in the training set for the top six descriptors in the model plotted against the descriptor values. Locally estimated scatterplot smoothing (LOESS) curves are overlaid on the descriptor observations (black dots). The midpoint in each curve indicates the cutoff value from where the prediction changes the direction. Positive SHAP values are associated with druggable and negative SHAP values with less druggable binding sites. The plots for the reaming descriptors are displayed in Figure S3.

The influence of the descriptors on the model was evaluated with the help of Shapley Additive Explanation (SHAP) values as implemented in the SHAPforxgboost package.^38−40^ The same package was also used to make Figure 2 and Figure S3. SHAP values describe the importance of each descriptor for the model output taking into account the interactions with other descriptors. Each descriptor for each data point (here, a particular binding site) is assigned a positive or negative SHAP value describing the contribution of the descriptor to the model output (here, druggable or less druggable) for that data point. The mean SHAP value formed by all SHAP values for a descriptor for the entire data set indicates the importance of the descriptor for the model (the larger the absolute mean SHAP value, the more important the descriptor). For DrugPred_RNA, positive SHAP values imply a high druggability probability, while negative SHAP values imply a low druggability probability. Further, by plotting the individual SHAP values for a descriptor against the descriptor values, it becomes evident which descriptor values contribute positively or negatively to the model. The sum of the SHAP values of all descriptors for a single data point indicates the direction of the prediction for that data point. Descriptors included in the final model were chosen by iteratively removing the least impactful descriptors until the predictive performance of the model was negatively affected. To further assess the robustness of the final model (called DrugPred_RNA), leave-one-out cross-validation was carried out, yielding a training and testing error of 0.00342 and 0.127, respectively.

In addition, accuracy precision and recall values of the models were calculated using eqs 1–3 with true positives (*tp*) and true negatives (*tn*) being the number of correctly classified binding sites and false positives (*fp*) and false negatives (*tn*) being the number of wrongly classified binding sites.
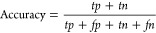
1
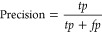
2
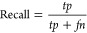
3

### Assembly of the Data Set with RNA Binding Sites

We selected RNA structures for druggability assessment by querying the PDB for structures containing only RNA and ligands (accessed November 2019). In addition, the PDB was searched for entries containing ligands and the keyword ″riboswitch″ to include structures that were excluded in the first query due to the presence of proteins. In total, this yielded 1084 structures. Subsequently, all structures that contained only ligands that were detergents, buffer salts, or crystallization components were filtered out, reducing the data set to 427 unique entries (Table 1, see the supplementary material for three-letter codes of rejected ligands). If a crystal structure contained several instances of the same ligand, only the first instance was retained. In addition, all metal ions and water molecules were deleted (for a list of metal abbreviations, see the supplementary material). This resulted in 465 distinct binding sites spanning 224 unique ligands. A second variant of this set was also prepared. In this variant, only pockets with metal ions that were not more than 5 Å away from a ligand atom were retained. If a binding site contained several metal ions, several copies of the binding sites, each of them containing one of the metal ions, were prepared. This variant contained 343 entries. In the following, the first variant is called the metal-free and the second variant the metal-containing set. Further, a data set containing ligand binding sites in ribosome crystal structures was compiled by querying the PDB for structures that contained ″ribosome″ as a keyword. These structures were treated as described above. In addition, the ligands were visually inspected to remove buffer components that had slipped the filter rules. This resulted in 613 binding sites in the metal-free ribosome set and 546 in the metal-containing set spanning 217 unique ligands.

**Table 1:** Data Sets of RNA and Ribosomal Binding Sites for Assessing DrugPred_RNA

	RNA-only set (metal-free/metal-containing set)	ribosome set (metal-free/metal-containing set)
unique PDB IDs	427	497
binding sites containing small molecule ligands	465/343	613/546
unique ligands	224	217
druggable entries	172/126	224/141

The binding site regions were carved out of the original CIF files by keeping all RNA residues with at least one atom within 15 Å of the ligand and potentially metal ions as described for the NRDLD set and subjected to descriptor calculations.

### Determination of Overall Sequence Similarity and Binding Site Similarity

To investigate the robustness of DrugPred_RNA toward changes in the binding site composition or conformation, binding sites were grouped into families based on overall sequence similarity and binding site similarity. For the grouping based on overall sequence similarity, the chains were aligned pairwise using BioPython’s pairwise2 global alignment function and the sequence similarity was calculated. If this value was >98%, the structures were assigned to the same family. For clustering based on binding site similarity, the binding site sequence of each pocket was generated by including all residues that contained at least one binding site atom in ascending order, while for modified nucleic residues, the name of the corresponding unmodified residue was used (see the supplementary material for a list of residue IDs for modified residues). Subsequently, all binding site sequences were aligned as described above. If the sequence similarity was >85%, the pockets were assigned to the same family.

### Consensus Scoring

As done previously, the consensus of the druggability predictions within each family of sequences (C) was calculated using the following formula:
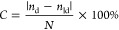
where *n*_d_ is the number of druggable binding sites within the family, *n*_ld_ is the number of less druggable binding sites, and *N* is the total number of family members.^23^ Thus, 100% consensus would be obtained if all pockets in one family were predicted to belong to the same class (druggable or less druggable) and 0% if one half of the pockets was predicted to belong to one class and the other half to the other class.

### Calculation of Ligand Properties

Physicochemical properties of the ligands in the RNA sets (Table 1) were calculated using RDKit. Further, the drug-likeness of ligands was estimated using the quantitative estimate of drug-likeness (QED) score as implemented in RDKit using average descriptor weights.^41^ This score weighs multiple molecular features (e.g., molecular weight, number of hydrogen bond donors or acceptors, polar surface area, and presence of unwanted functionalities) into one single unitless score, which ranges from 0 (undesirable) to 1 (desirable). Although this metric does not provide a clear cutoff to distinguish “desirable” from “undesirable” compounds, the authors denoted a mean score of 0.67 for attractive compounds, 0.49 for less attractive compounds, and 0.34 for too complex and unattractive compounds. Accordingly, we classified compounds with a QED score ≥ 0.67 as drug-like, those with a QED score ≤ 0.49 as less drug-like, and those with a score in between as moderate drug-like.

## Results and Discussion

### Construction of DrugPred_RNA

Compared to protein data, there are very little data about ligands binding to RNA, and a data set of sufficient size composed of druggable or less druggable RNA binding sites to train a druggability predictor could not be compiled. Therefore, we opted to predict the druggability of RNA binding sites by training a descriptor on protein binding sites and to subsequently apply it to the prediction of RNA pockets. This approach required that only descriptors that can be calculated for both protein and RNA binding sites were used. This was not the case for our previously derived DrugPred model, as it contained the two descriptors “relative occurrence of hydrophobic amino acid” and “hydrophobicity indices of the amino acids”.^22^ Thus, a modified DrugPred model, termed DrugPred_RNA, was derived. As a training and test set, our NRDLD set of druggable and less druggable binding sites with the most recent modifications was used.^22,23^ For all 110 binding sites in the NRDLD, 23 descriptors describing the size, shape, and polarity were calculated (Table S1). Subsequently, the data set was divided into a training and test set as done previously^23^ to train and evaluate a predictor. For DrugPred and DrugPred 2.0, partial least squares-discriminant analysis (PLS-DA) was used to model the data. However, using only protein-independent descriptors with PLS-DA resulted in worse predictions (data not shown). Therefore, we retreated to decision tree modeling based on XGBoost.^31^ To avoid overfitting, the maximum depth of trees was limited to 3 and the early stopping option was used (Figure S1). In an iterative process, weak descriptors as judged by SHAP values were removed until the predictive performance of the model was negatively affected. With the final model, termed DrugPred_RNA, of the 75 binding sites in the training set, 1 druggable pocket was misclassified as less druggable, and of the 35 binding sites in the validation set, 4 were misclassified (2 false positives and 2 false negatives), leading to accuracy, precision, and recall values between 0.86 and 1.00 (Table 2 and Figure S2). With DrugPred_RNA, the performance for the training set was or slightly improved compared to DrugPred 2.0, while the performance for the test set was slightly worse.

**Table 2:** Performance of DrugPred_RNA and DrugPred 2.0 on the Training and Test Set of the NRDLD

	training set [druggable/less druggable]		test set [druggable/less druggable]	
	DrugPred_RNA	DrugPred 2.0	DrugPred_RNA	DrugPred 2.0
accuracy	0.99	0.91	0.91	0.94
precision	1.00/0.97	0.92/0.89	0.95/0.86	0.95/0.93
recall	0.98/1.00	0.94/0.86	0.91/0.92	0.95/0.93

The final DrugPred_RNA predictor was based on 12 descriptors (Figure 2A and Table S1). According to the SHAP values, the two most important descriptors were the relative polar surface area (*psa_r*, absolute mean SHAP value = 1.46) and the fraction of hydrophobic binding site atoms (*fr_hpb_atoms,* absolute mean SHAP value = 0.63), which both describe the polarity of the binding site. As expected, druggable binding sites were less polar than less druggable sites (Figure 2B and Figure S3)*.* Both the high-ranking descriptor *fr_buried_sl_atoms* (absolute mean SHAP value = 0.34) and the less important descriptor *sa_vol_*r (absolute mean SHAP value = 0.09) encode how compact a pocket is, with less druggable pockets being more shallow (lower descriptor values for *fr_buried_sl_atoms* and higher values for *sa_vol_r*) than druggable ones. Further, two descriptors for the solvent accessibility of the pocket (*exp_sl_sa*, absolute mean SHAP value = 0.22 and *sl_bs_r*, 0.19) were included in the final model. Here, it was found that druggable binding sites were less solvent accessible than less druggable ones. The descriptor *hsa* was also found to be among the more important ones (absolute mean SHAP value *=* 0.30). This descriptor describes the size of the surface area of hydrophobic binding site atoms and correlates roughly with the size of the pocket. Other descriptors describing the size of the pocket were also included in the model but had less influence on the predictions (*no_bs_atoms*, absolute mean SHAP value = 0.17; *no_sl_atoms*, absolute mean SHAP value = 0.20). In agreement with previous findings, druggable pockets were larger and more hydrophobic than less druggable ones. The descriptors *InertialShapeFactor*, *SpherocityIndex*, and *PMI3* describing the shape of the superligand as a negative print of the binding site were also included in the final model. Pockets with a superligands with a larger third moment of inertia (*PMI3*, absolute mean SHAP value = 0.27) and that were less spherical (*SpherocityIndex*, absolute mean SHAP value = 0.08; *InertialShapeFactor*, absolute mean SHAP value = 0.08) were more likely to be assessed as druggable, albeit the latter two descriptors were determined to be less important.

### Druggability Predictions for RNA-Containing Binding Sites

Encouraged by the good performance of DrugPred_RNA on the NRDLD, we proceeded with druggability predictions for RNA and ribosomal binding sites. Using the PDB, we compiled two data sets for this purpose, one containing RNA-only binding sites and one with ribosome binding sites that, in addition to ribosomal RNA, could also contain ribosomal proteins. As binding sites, we considered all pockets that contained a ligand that is not a common crystallization buffer component. If a binding site contained metal ions within 5 Å of the ligand, several copies of the binding sites, each of them containing one of the metal ions in addition to the metal-free pocket, were prepared. In total, the RNA-only binding site set was composed of 427 unique PDB IDs spanning 465 binding sites in the metal-free and 343 in the metal-containing subset (Table 1). A total of 224 different ligands were found in these pockets. The ribosomal binding site set was prepared in a similar fashion, resulting in 497 unique PDB IDs with 613 pockets in the metal-free and 546 in the metal-containing subset containing in total 217 different ligands.

The ligands in both sets spanned a wide range of physicochemical properties (Figure S4). Generally, the descriptor space for ribosomal and RNA-only ligands overlapped. However, the medians of the molecular weight, number of hydrogen-bond acceptors and donors, rotatable bonds, clogP, and fraction of sp3 carbon atoms were higher among the ligands in the ribosomal set compared to the ligands in the RNA-only set. In contrast, the median of the number of aromatic rings was higher in the RNA-only set, while the median of the number of rings was the same in both sets.

**Figure 3:**
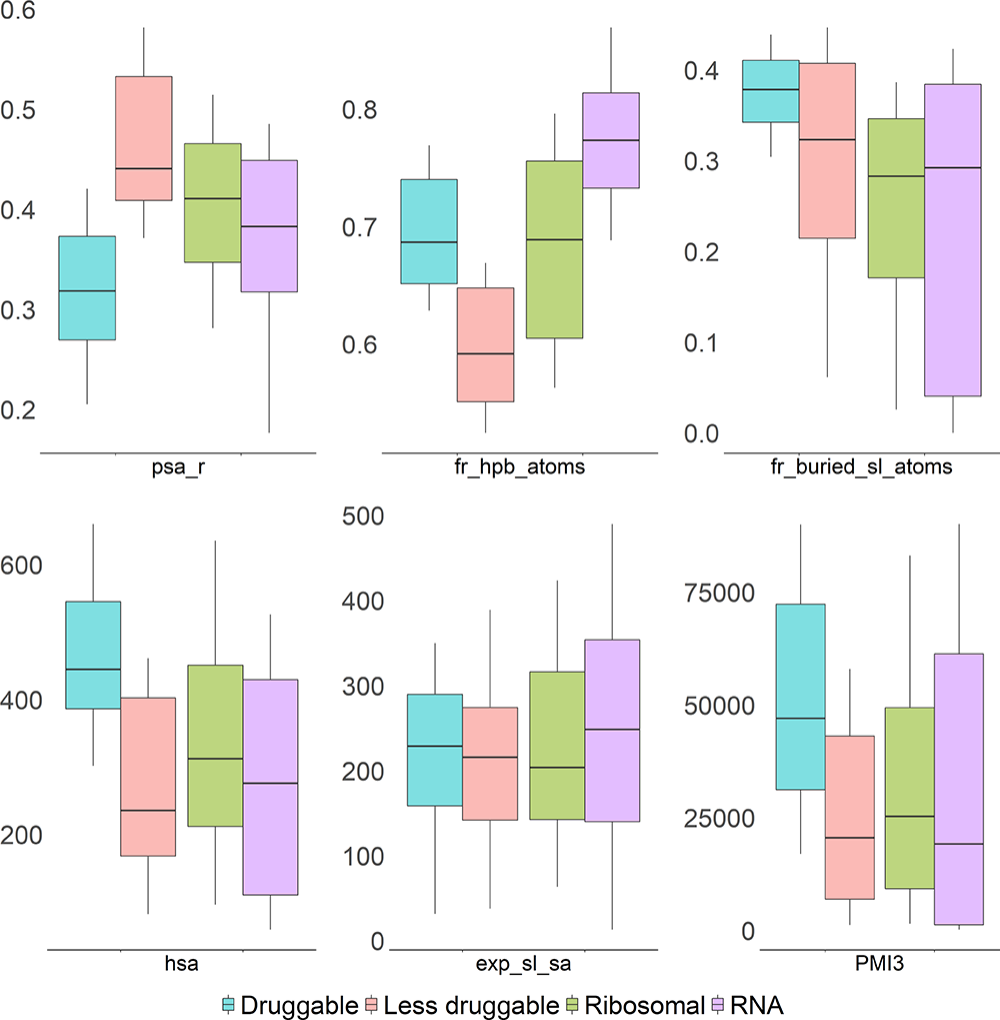
Boxplots showing the distribution of the six highest-ranking descriptors in the DrugPred_RNA model for the druggable and less druggable protein binding sites in the NRDLD set and the RNA binding sites in the RNA sets. The lower and upper hinges of the boxes represent the 25th and 75th percentiles of the data, and the whiskers extend to the bottom 10th and upper 90th percentile. The line inside the boxes marks the median value. The plots for the reaming descriptors are displayed in Figure S5.

Next, descriptors for all pockets in the sets were calculated and compared to the descriptors of the NRDLD set (Figure 3 and Figure S5). In general, the descriptors for the druggable protein binding sites were more narrowly distributed than those for less druggable protein binding sites or the RNA pockets. Both RNA sets (ribosomal and RNA-only) had binding sites for which the descriptor values were in the same range as those found for druggable protein pockets.

Subsequently, the druggability of the pockets in all sets was predicted. In the RNA data set, 36% of the binding pockets (metal-containing and metal-free combined) were predicted to be druggable, while in the ribosomal data set, 31% of the pockets were predicted to be druggable (see the supplementary material for individual predictions for all pockets).

To assess the impact of metal ions on the druggability prediction, we compared the predictions of metal-free and metal-containing versions of same parent pocket. In both sets, for the majority of the cases (90% in the RNA-only set and 83% in the ribosome set), no change in the prediction outcome was found. Accordingly, metal ions had only a minor influence on the predictions. In the following, we therefore only present data for pockets that were stripped of metal ions.

### Criteria for the Assessment of Druggability Predictions for RNA Binding Sites

Next, the quality of the predictions of DrugPred_RNA for RNA binding sites was evaluated. No benchmark set for the evaluation of RNA druggability predictions is available in the public domain. Therefore, we evaluated the performance of DrugPred_RNA on the above described RNA sets based on the following criteria: (1) the agreement of the predictions with how one would judge the druggability based on visual inspection of the binding site and the properties and affinities of the known ligands, (2) the extent to which binding sites that efficiently bind drug-like ligands were predicted to be druggable, and (3) the robustness of the predictions with respect to substitutions and conformational changes in the binding sites. In this context, we considered a ligand to bind tightly to a binding site if it had a ligand efficiency (the binding energy normalized by the number of heavy atoms, LE) at least close to 0.30 kcal·mol^–1^·heavy atom^–1^, which translates to low nanomolar binding affinities of compounds with a molecular weight of maximum 500 Da under the assumption that the ligand efficiency stays at its best constant during optimization.^42^ Such a measure of tight binding takes into account that a small ligand with a weak affinity can potentially be optimized to a larger, more potent ligand.

### Evaluation of the Performance of DrugPred_RNA Based on Visual Inspection of Binding Sites and Properties of Bound Ligands

**Figure 4:**
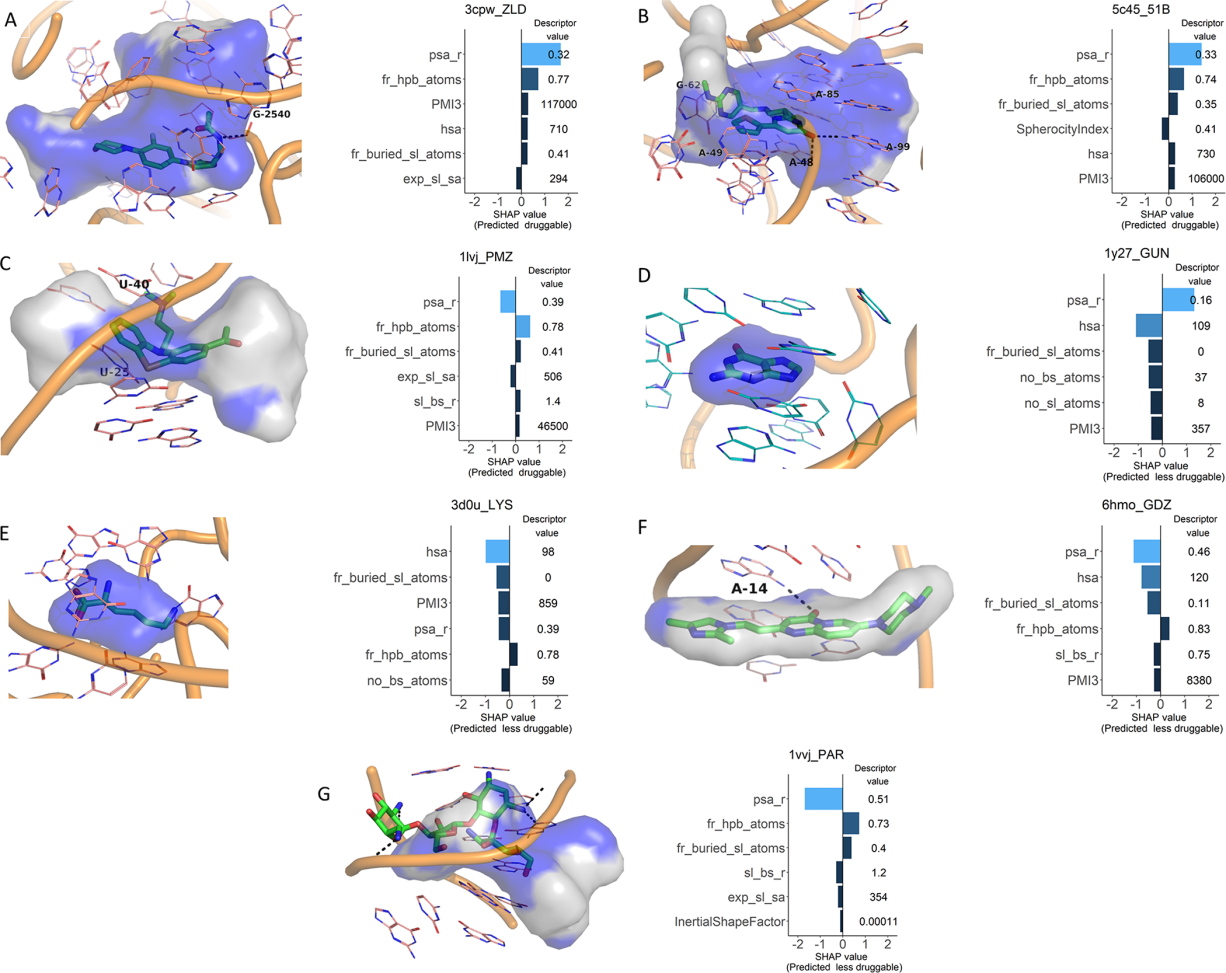
Evaluation of the performance of DrugPred_RNA based on selected examples. The RNA backbones are shown as orange tubes, nucleobases as thin sticks with carbon atoms colored pink, and ligands as thick sticks with carbon atoms in green. The surface of the superligand created by DrugPred_RNA as a negative print of the pocket is shown as blobs with the solvent exposed surface area colored gray and the remaining surface area colored blue. Hydrogen bonds are indicated as dotted black lines. For each pocket, the individual SHAP values for the six most important descriptors together with the descriptor values are also displayed. The SHAP value plots are labeled with the PDB IDs of the receptors and the three-letter codes of the ligands found in each pocket. (A) The binding site of linezolid in the 50S ribosomal subunit. (B) Ribocil bound to the FMN riboswitch. (C) TAR RNA complexed with acetylpromazine. (D) Guanine bound to the guanine riboswitch. (E) Lysine in the binding site of the lysine riboswitch. (F) Splicing site complexed with a splicing site modifier. (G) Paromomycin bound to a bacterial ribosome site.

In the absence of a benchmarking set to assess the performance of RNA druggability predictions, we chose a few examples from the RNA sets for a first validation of the predictions. The examples were selected to have published affinity data for at least the co-crystallized ligand, cover different RNA classes, and have different prediction outcomes. The list included two ribosomal binding sites (Figure 4A,G); the FMN, guanine, and lysine riboswitches (Figure 4B,D,E); TAR RNA (Figure 4C); and a splicing site (Figure 4F). The binding pockets of the selected examples were visually inspected. Pockets that were large enough to accommodate a drug-sized ligand, that were partially buried, and for which a drug-like ligand binding with high ligand efficiency was known were judged to be druggable, whereas the remaining pockets were judged to be less druggable. This resulted in the linezolid binding site in the 50S ribosomal subunit (Figure 4A), the FMN riboswitch binding site (Figure 4B), and the TAR RNA binding site (Figure 4C) to be manually assigned as druggable and the binding sites in the guanine and lysine riboswitch (Figure 4D,E) as well as the splicing site (Figure 4F) to be assigned as less druggable. (More details about the manual assignment of the binding sites and the DrugPred_RNA predictions can be found in the supplementary material.) The predictions obtained by DrugPred_RNA (Figure 4, right panels) agreed with the manual assignment for all pockets.

### Druggability Predictions of RNA Pockets Binding to Drug-like Ligands

In the next step, we investigated which prediction pockets in our RNA test sets obtained that contained drug-like ligands. By definition, a pocket binding potently to a drug-like ligand is considered to be druggable.^17,18^ We therefore expect from a well-performing druggability predictor that pockets binding to drug-like ligands are predicted as druggable. However, a pocket binding to a non-drug-like ligand is not necessarily less druggable as it could be that a potent drug-like ligand has simply not yet been found. This is a particular concern when working with RNA binding sites, as ligand space is typically much less explored than for protein binding sites and, in addition, the available ligand information cannot easily be mined using computational methods. Accordingly, validating structure-based druggability predictions based on pockets binding to non-drug-like ligands would be associated with a large uncertainty, and we therefore abstained from discussing predictions obtained for these pockets.

In total, the RNA sets contained 331 unique ligands with 22 of them having a QED score ≥ 0.67. Four of these ligands were found in the binding site of the preQ1 riboswitch. Upon closer inspection of these pockets, it became evident that some of the bases in these structures were not resolved (e.g., the residues 13–15 in PDB ID 6e1t and the residues 13–14 in PDB ID 6e1v). These pockets were therefore not further considered. Out of the remaining ligands, 12 (67%) were found in binding sites assessed by DrugPred_RNA as druggable (Table 3) and 6 (23%) in binding sites assessed to be less druggable (Table 4). As only 37% of all metal-free binding sites were predicted to be druggable, the drug-like ligands were clearly enriched in druggable binding sites.

**Table 3:** Drug-like Ligands (QED ≥ 0.67) Found in RNA Binding Sites Predicted to Be Druggable

ligand ID	PDB ID	receptor name	QED score	*K*_d_ [nM]	LE [kcal·mol^–1^·heavy atom^–1^]
RNA data set					
MGR	1q8n	malachite green aptamer	0.76	800^43^	0.34
6YG	5kx9	FMN riboswitch	0.69	13.4^44^	0.41
L8H	2l8h	HIV-1 TAR RNA	0.67	NA^a^^,^^45^	
PMZ	1lvj	HIV-1 TAR RNA	0.85	27,000^46^	0.22
Ribosomal data set					
917	5v7q	50S ribosomal subunit	0.94	700^47^	0.39
ZLD	3cpw	50S ribosomal subunit	0.89	20,000^48^	0.27
G6M	6ddg	50S ribosomal subunit	0.79	2600^49^	0.31
3HE	4u3u	80S ribosome	0.76	140^51^	0.48
G6V	6ddd	50S ribosomal subunit	0.76	2600^49^	0.30
ANM	3 cc4	50s ribosomal subunit	0.78	20,000^52^	0.34
HN8	5on6	80S ribosome	0.71	NA^a^	
3 K8	4u55	80S ribosome	0.71	39	0.32

a Binding affinity unknown.

**Table 4:** Drug-like Ligands (QED ≥ 0.67) Found in RNA Binding Sites Predicted to Be Less Druggable

ligand ID	PDB code	receptor name	QED	*K*_D_ [nM]	LE [kcal·mol^–1^·heavy atom^–1^]
RNA data set					
VIB	4nyg	TPP riboswitch	0.79	1500^53^	0.45
2QC	4nyb	TPP riboswitch	0.77	103,000^53^	0.43
0EC	2lwk	influenza A virus RNA promoter region	0.86	50,000^54^	0.29
1TU	5ob3	Spinach aptamer	0.85	530^55^	0.49
218	2hop	TPP riboswitch	0.77	6000^56^	0.38
Ribosomal data set					
TRP	4v6o	tryptophan-sensing ribosomal site	0.67	NA^a^	

a Binding affinity unknown.

For 10 out of the 12 drug-like ligands binding to pockets predicted to be druggable, we could find binding data in the literature (Table 3). Based on these data, eight ligands bind efficiently to their target with LEs > 0.30 kcal·mol^–1^·heavy atom^–1^, hinting that these pockets are indeed druggable. The two remaining ligands were linezolid with the 50S ribosomal subunit as target and acetylpromazine binding to HIV-1 TAR RNA (Figure 4A,C). Based on manual assignment (see the supplementary material), these pockets also appear to be druggable. Thus, all predictions for the pockets binding to the 10 drug-like ligands with accessible binding data appear to be valid.

**Figure 5:**
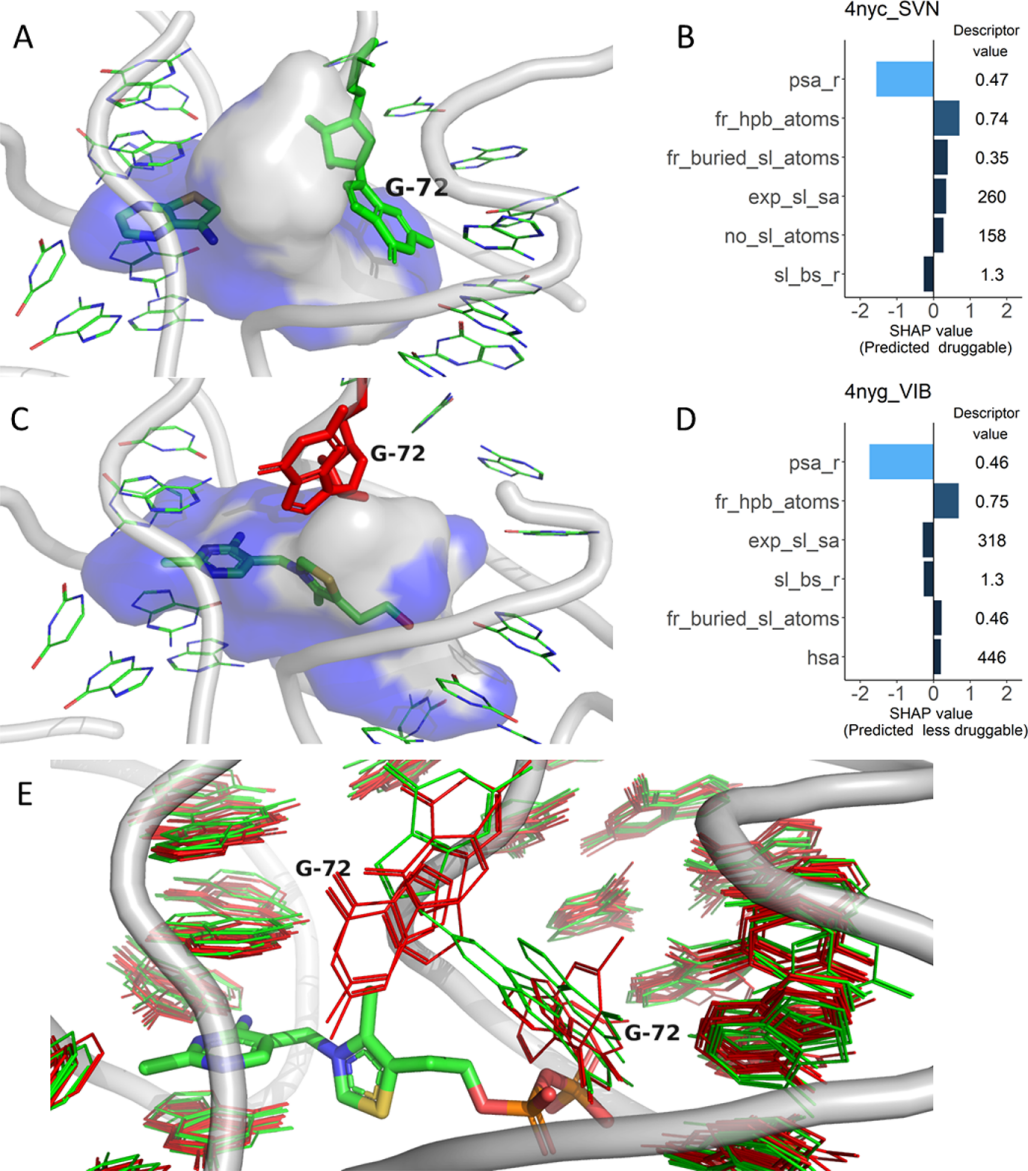
Druggability predictions for TPP riboswitch binding sites, with the flexible residue G72 highlighted. The surface of the superligand created by DrugPred_RNA as a negative print of the pocket is shown as a blob with the solvent exposed surface area colored gray and the remaining area colored blue. For the pockets shown in (A) and (B), the individual SHAP values for the six most important descriptors are shown together with their descriptor values. The SHAP plots are labeled with the PDB IDs of the receptors and three-letter codes of the ligands found in each pocket (B, D). (A) TPP riboswitch binding site (PDB ID 4nyc) in complex with a fragment screening hit (green sticks). (C) TPP riboswitch binding site (PDB ID 4nyg) in complex with thiamine. (E) Superposition of all *E. coli* TPP riboswitch binding sites in the RNA-only set. Entries predicted to be druggable are colored green, and those predicted to be less druggable are colored red. For clarity, only the backbone (gray tube) from PDB entry 4nyc is shown. The conformation of the residue G72 influences the prediction.

**Figure 6:**
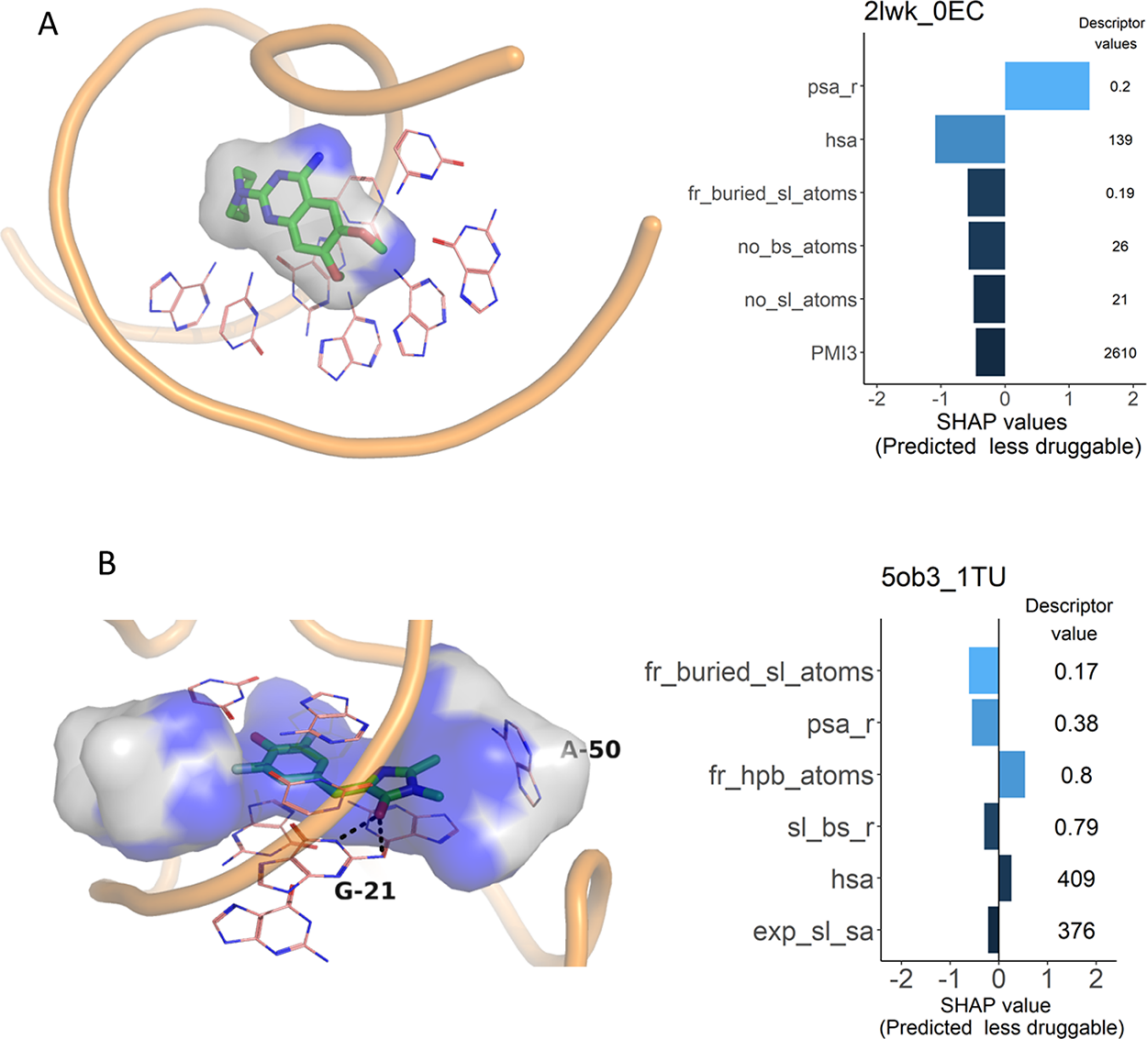
RNA binding sites predicted to be less druggable but binding drug-like ligands. The surface of the superligand created by DrugPred_RNA as a negative print of the pocket is shown as a blob with the solvent exposed surface area colored gray and the remaining surface area colored blue. For each pocket, the individual SHAP values for the six most important descriptors together with the descriptor values are also displayed. The SHAP value plots are labeled with the PDB IDs of the receptors and the three-letter codes of the ligands found in each pocket. (A) Binder (green) of influenza A promoter region (PDB ID 2lwk). (B) The Spinach aptamer (PDB ID 5ob3) bound to the dye DFHBI (green).

On the other hand, six drug-like ligands were found in pockets predicted to be less druggable (Table 4). For five of them, we could retrieve affinity data in the literature, and all of these bind rather efficiently to their targets (LE ≥ 0.29 kcal·mol^–1^·heavy atom^–1^). Three of these ligands are fragments binding to the TPP riboswitch, one is a ligand binding the influenza A virus promoter region, and one a ligand of the Spinach aptamer. Several examples of the TPP riboswitch binding site were contained in the RNA-only set (Figure 5). The pockets differ mainly in the conformation of G72 (Figure 5E), but in all cases, the pocket is rather large and partially buried (Figure 5A-D). The pockets with G72 in one of the conformations were predicted to be druggable (Figure 5A, B), while pockets with G72 in the alternative conformation (Figure 5C, D), including the ones binding the drug-like fragments, were predicted to be less druggable. Based on the structures, discussed in more detail below, it is not obvious why the latter TPP riboswitch binding sites should be less druggable. These predictions can therefore be considered false negative. The drug-like ligand of the influenza A promoter region sits on the surface of the RNA molecule and is almost entirely solvent exposed (Figure 6A). It is highly unusual that a ligand with such a binding mode binds that efficiently (LE = 0.29 kcal·mol^–1^·heavy atom^–1^). However, the structure of the complex has been determined by NMR, and it is possible that the resolution of the structure is not accurate enough to reveal the details of the binding mode.^54^ The small molecule dye, DFHBI, is bound deep into the solvent-excluded part of the pocket in the Spinach aptamer, forming pi-stacking interactions and hydrogen bonds with the surrounding residues (Figure 6B). Considering the drug-likeness of the ligand together with its efficient binding and its binding mode, the prediction for this pocket by DrugPred_RNA is likely wrong.

Taken together, the druggability predictions for the pockets predicted to be druggable and binding to drug-like ligands appeared to be correct, while four out of five pockets predicted to be less druggable are likely false negatives (three of the false-negative predictions are pockets arising from the same target but are bound to different ligands). These could suggest that DrugPred_RNA has a larger tendency to misclassify druggable binding sites as less druggable than vice versa, as already observed for the NRDLD test set (precision = 0.95 for druggable pockets vs 0.86 for less druggable pockets, Table 2). However, the investigated data set was too small to conclude firmly on this.

### Assessment of the Robustness of the Druggability Predictions

Finally, we assessed the robustness of the predictions with respect to small changes of the conformation or base composition of the binding sites. To this end, the pockets in the RNA sets were grouped based on two different measures: overall sequence similarity and binding site similarity. For overall sequence similarity and for binding site similarity, a cutoff of 98% and 85%, respectively, was used for grouping similar sequences into the same family. The lower cutoff value for binding site similarity was chosen to allow for some variation in the binding sites considering the low number of binding site residues (on average, about 15 residues for the RNA data set and 47 for the ribosomal data set). Grouping based on overall sequence similarity was only carried out for the RNA subset as the ribosome structures contain several pockets and thus grouping based on sequence similarity would have resulted in different pockets in the same family. This procedure resulted in 57 families for the RNA-only set based on overall sequence similarity and 46 families based on binding site similarity (Tables S2 and S3). For the ribosome set, 52 families were found based on binding site similarity (Table S4). Subsequently, the consensus of the predictions for each family was calculated. In the RNA-only set, a consensus of 100% was obtained for 79% of the families grouped based on overall sequence similarity and for 74% of the families grouped based on binding site similarity. In the ribosome set, for 75% of the families, all members obtained the same druggability prediction. Thus, in most cases, using different crystal structures of the same or a related pocket did not change the outcome of the prediction.

Next, selected families were more closely investigated to obtain an understanding as to which binding site changes caused a low consensus score. For this purpose, the TPP and ZTP riboswitch families (Table S2) as well as the neomycin binding site of bacterial ribosome were chosen (Table S4) because they had a low consensus for the predictions, they had more than two members, all structures in these families were determined using X-ray crystallography, all binding site residues were resolved, and they contained only naturally occurring RNA.

The TPP riboswitch family that contained pockets from 16 distinct PDB entries when clustered based on binding site similarity obtained a low consensus score of 12.5% with the majority of the pockets predicted as less druggable (Table S2). Superimposing the pockets, it became evident that there is some plasticity in the binding site (Figure 5E). One guanine residue (G72 in the *E. coli* TPP riboswitch) can adopt several conformations depending on the bound ligand, leading to considerably different superligands (Figure 5A,C). Consequently, the pockets differ in compactness (*fr_buried_sl_atoms*, *sl_bs_r*) and solvent exposure (*exp_sl_sa*), leading to different prediction outcomes. However, based on the structures and the affinity of the bound ligands, both binding sites appear to be druggable and thus, in this case, some of the predictions are likely wrong.

**Figure 7:**
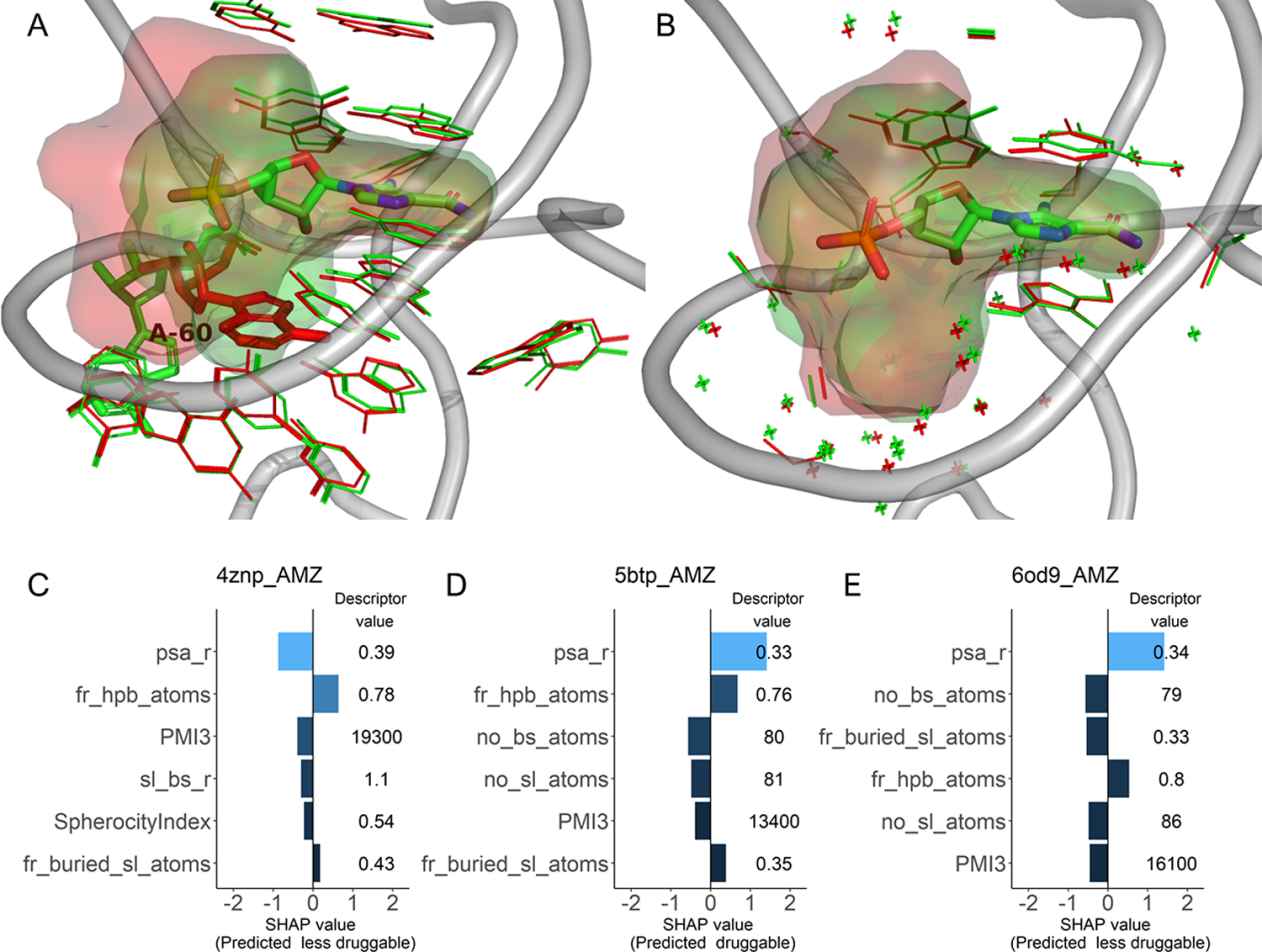
Superposition of the ZNP riboswitch binding sites bound to ZNP (thick sticks with green carbon atoms). The superligands created by DrugPred_RNA are shown as blobs. For clarity, only the backbone from 5btp is shown. (A) Superposition of the pockets of the structures with the PDB IDs 4znp (red, less druggable) and 5btp (green, druggable). The entire residues forming the binding sites are shown. The residue A60 is adopting two different conformations. (B) Superposition of the pockets of the structures with the PDB IDs 5btp (green, druggable) and 6od9 (red, less druggable). For clarity, only the atoms that DrugPred_RNA predicted to be in contact with the superligand are shown (thin sticks/crosses). (C, D, E) Individual SHAP values for the six most important descriptors for the displayed binding sites together with the descriptor values.

Another family with a low consensus is the ZTP riboswitch (33.3%), with the majority of the pockets predicted to be less druggable (Table S2). The three entries in the family are all bound to the same ligand, ZMP (aminoimidazole 4-carboxamide ribonucleotide), which is poorly drug-like (QED = 0.39). Superposition of the druggable pocket with the less druggable pockets revealed that one of the less druggable pockets has a clearly different conformation of the residue A60 resulting in very different superligands for the druggable and one of the less druggable pockets and thus different predictions (Figure 7A,C,D). The second less druggable pocket has nearly the same conformation as the druggable pocket (Figure 7B). In this case, subtle conformational changes were enough to obtain a slightly different superligand that in turn resulted in a switch of the prediction despite the descriptors with top six highest SHAP values being almost identical (Figure 7D,E).

**Figure 8:**
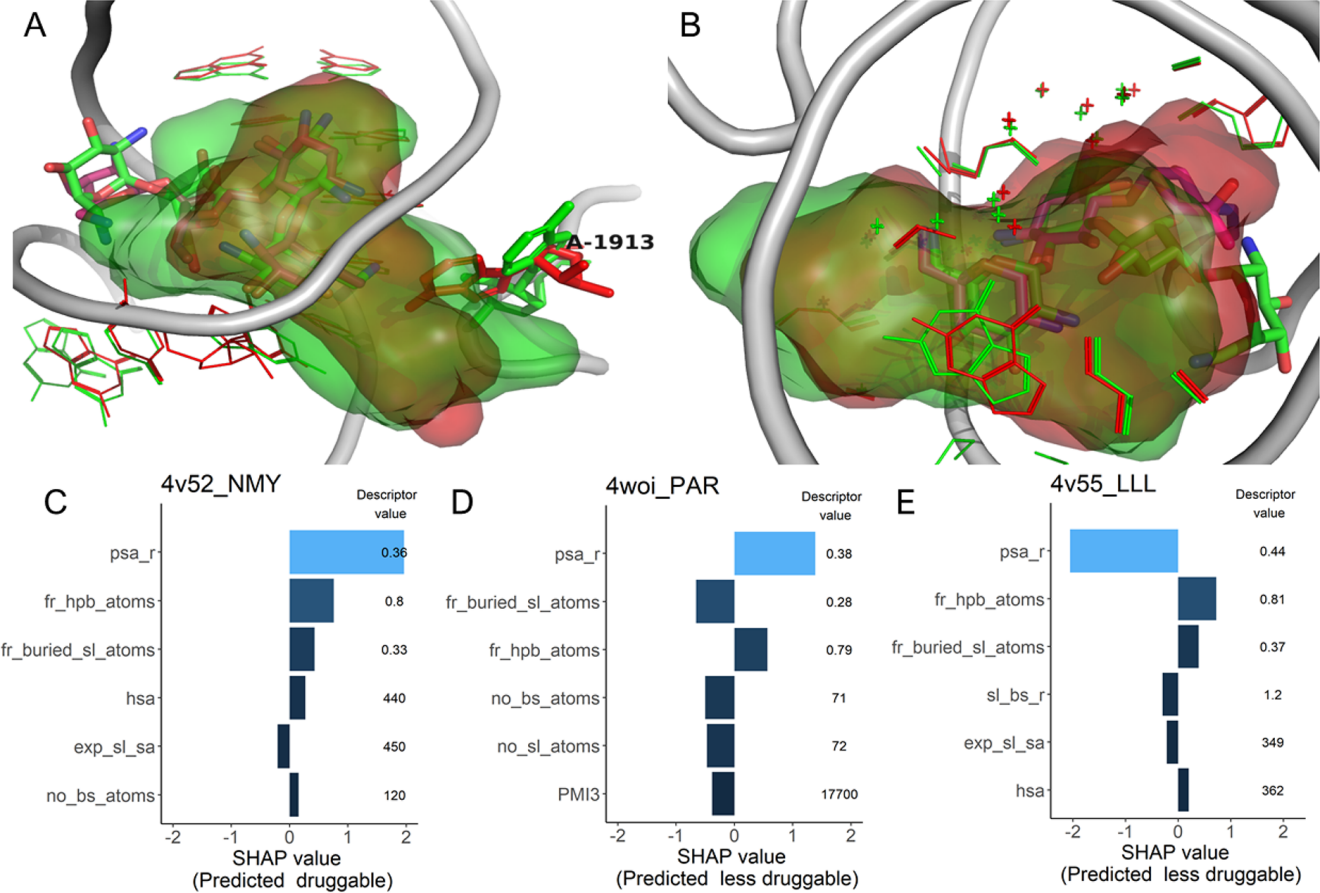
The neomycin ribosomal binding site family. (A) Superposition of the neomycin- (PDB ID 4v52, green) and paromomycin- (PDB ID 4woi, magenta and red) containing ribosomal binding sites. The backbone (taken from PDB ID 4v52) is shown as thick gray tube, and the superligands created by DrugPred_RNA are shown as blobs (green: 4v52, red: 4woi). A1913 is highlighted with thick lines. (B) Superposition of the neomycin- (green, thick sticks) and gentamicin (magenta, thick sticks)-containing binding sites (PDB IDs 4v52, 4v55) showing only atoms (thin lines, crosses) in direct contact with the superligands (green blob, 4v52, red blob 4v55). (C, D, E) Individual SHAP values for the six most important descriptors together with the descriptor values. The label denotes the PDB ID of the structure followed by the three-letter code of the ligand.

The family containing the neomycin binding site of bacterial ribosome obtained a consensus score of 0% based on clustering by binding site similarity (Table S4). The two druggable entries in this family were bound to neomycin (PDB IDs 4v52 and 4v57), while the two less druggable entries were bound to paromomycin (4woi) and gentamicin (4v55). Compared to the neomycin-containing structures, A1913 is rotated in 4woi, leading to a very different shape and size of the pocket with a different prediction outcome (Figure 8A,C,D). The structural differences between the pocket in 4v55 and the druggable sites are less pronounced but nevertheless sufficient to make the pocket more polar and thus less druggable (Figure 8B,E).

In summary, in most of the cases (≥74%, depending on the set), binding sites from related structures obtained the same druggability predictions. However, there were also examples as discussed above where this was not the case. In some of the illustrated examples, a conformational change of a residue in the binding site led to a clearly differently shaped pocket, and it was easily comprehensible why this could influence the predictions (Figures 5, 7A, and 8A). In other cases, the conformational changes were more subtle but nevertheless, in their sum, led to different predictions (Figures 7B and 8B). Thus, it appears to be advisable to score more than one example of a binding site if available to obtain reliable results.

## Conclusions

RNA is an emerging target for drug discovery.^3−6^ However, like for proteins, not all RNA binding sites are equally suited to be addressed with conventional drug-like ligands. We have developed the structure-based druggability predictor DrugPred_RNA to identify pockets that are primed to potently bind such ligands. Due to the paucity of annotated RNA binding sites, the predictor was trained on a set of protein pockets, albeit containing only descriptors that can be calculated for both RNA and protein binding sites. DrugPred_RNA performed comparably on the protein binding site set as our previous DrugPred 2.0 predictor trained with slightly different descriptors (Table 2). In addition, druggability predictions of DrugPred_RNA for all manually selected examples were in agreement with druggability assignments based on visual inspection and properties of bound ligands (Figure 4). When assessing the performance of DrugPred_RNA based on RNA-containing binding sites bound to drug-like ligands (Tables 3 and 4), all predictions for pockets predicted to be druggable and for which affinity data could be found were correct (Table 3), while for pockets predicted to be less druggable, four out of five predictions were likely wrong (Table 4). Overall, these data could suggest that DrugPred_RNA has a higher false positve rate for predicted less druggable binding sites than predicted druggable binding sites, Table 2, but the investigated RNA subset was too small to firmly conclude on this. Further, using different conformations of a binding site or pockets with a slightly different sequence composition could result in opposing druggability predictions (Tables S2–S4). The same was observed before for druggability predictions for proteins.^22,57^ In this study, both large and small conformation changes could influence the prediction outcome (Figures 5, 7, and 8). Nonetheless, for the majority of cases (≥74%, depending on the set), consistent predictions were obtained indicating that DrugPred_RNA is generally robust toward small changes in binding site conformations and compositions.

Compared to proteins, RNA binding sites are not well explored, and only limited ligand information is available. The combined metal-free RNA and ribosome binding site sets contained 1078 pockets (Table 1). Only 22 of them bound to a drug-like ligand, and for 18 of them, affinity data could be retrieved (Tables 3 and 4). In contrast, 396 pockets in the metal-free sets were predicted to be druggable by DrugPred_RNA based on their binding site properties (Table 1). This points to ample opportunities to develop drug-like RNA ligands. Interestingly, many riboswitches were found among the binding site families that were predicted to be druggable (Table S2). This finding underlines the notation that these promising targets for new antibiotics could be addressed with drug-like ligands.^3,12,21^ Further, also in the ribosomal binding site set, druggable pockets were contained (Table S4). These predictions can help to direct efforts when targeting the ribosome for the development of drugs to overcome the looming antibiotic crisis.^7,49^

**Figure 9:**
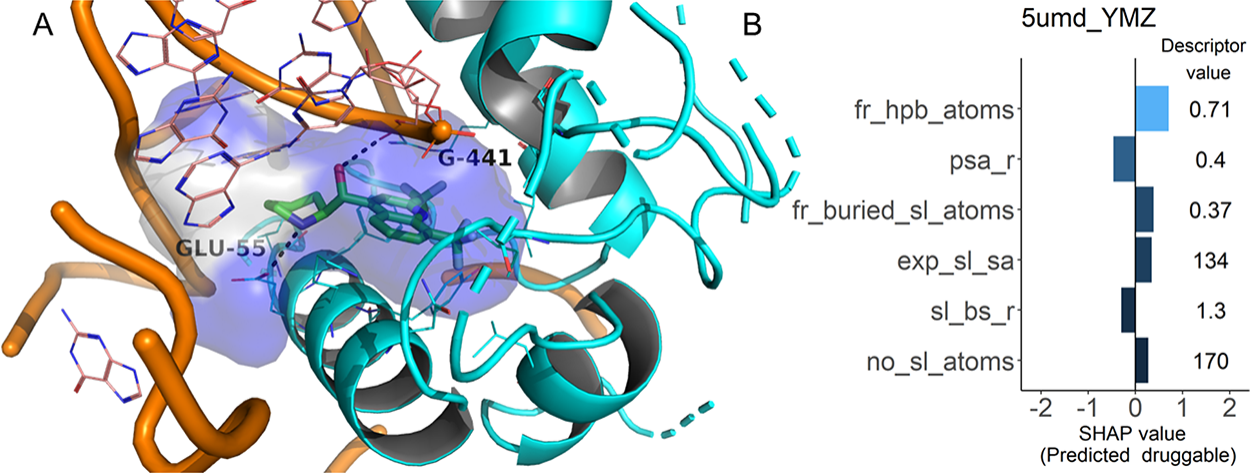
(A) Ribosomal binding site of mefloquine that is formed by amino acids (cyan) and bases (orange, PDB ID 5umd). The ligand mefloquine is shown as green sticks, while the surface of the superligand created by DrugPred_RNA as a negative print of the pocket is shown as a blob with the solvent exposed surface area colored gray and the remaining surface area colored blue. (B) Individual SHAP values for the six most important descriptors together with the descriptor values obtained by DrugPred_RNA.

Notably, as DrugPred_RNA was trained with descriptors that can be calculated for both RNA and protein binding sites, it can also be used to score pockets that are formed by both types of macromolecules. An example is a pocket in the protozoal 80S ribosomal site that highly efficiently (LE = 0.41 kcal·mol^–1^·heavy atom^–1^) binds to the drug-like molecule mefloquine (QED = 0.79) and was predicted to be druggable (Figure 9).^58^

To conclude, DrugPred_RNA is a promising tool for structure-based druggability predictions of RNA binding sites that can be used to prioritize targets and to decide if a target can be addressed with drug-like ligands or another area of chemical space has to be searched for potent ligands.

## Supporting Information Available

Information about manual druggability assignment of RNA binding sites, tables with more information about the descriptors (Table S1), and overviews of the binding site families and consensus scoring results (Tables S2–S4) as well as figures displaying the training and testing set accuracy error during the construction of DrugPred_RNA (Figure S1), druggability predictions with DrugPred_RNA for the NRDLD training and test set (Figure S2), individual SHAP values for each pocket in the training set for all descriptors in the model plotted against the descriptor values (Figure S3), as well as density plots for physicochemical properties of the ligands in the RNA sets (Figure S4) and binding site descriptors (Figure S5) (PDF)
ci1c00155_si_001.xlsx: Three-letter codes of ligands that were treated as buffer components (XLSX)
ci1c00155_si_002.xlsx: Metal abbreviations (XLSX)
ci1c00155_si_003.xlsx: List of commonly modified RNA residues (XLSX)
ci1c00155_si_004.xlsx: List of commonly modified RNA residues (XLSX)
ci1c00155_si_005.xlsx: Druggability predictions for all pockets in the ribosome and RNA-only sets (XLSX)
ci1c00155_si_006.xlsx: Molecular formula strings (XLSX)

## Notes

Only publicly available data have been used. The method section contains detailed descriptions on how the data sets were compiled. Subsets of the data are also attached as Supporting Information. The scripts to run DrugPred_RNA as command line application and instructions on how to use them are available from https://github.com/ruthbrenk/DrugPred_RNA. DOCK 3.6 and DelPhi, which is needed for DOCK, are licensed software that can be obtained from http://dock.compbio.ucsf.edu/ and http://honig.c2b2.columbia.edu/delphi.
