# Supplementary material for: DrugPred_RNA—A Tool for Structure-Based Druggability Predictions for RNA Binding Sites
Source: J Chem Inf Model. 2021 Jul 21;61(8):4068–81. doi: 10.1021/acs.jcim.1c00155 (PMC8389535; doi:10.1021/acs.jcim.1c00155)
Supplement: Supplementary file 7 — ci1c00155_si_007.pdf [file ci1c00155_si_007.pdf]

Supporting information

DrugPred_RNA – A tool for structure-based druggability predictions for RNA binding sites

Illimar Hugo Rekand and Ruth Brenk*

Department of Biomedicine, University of Bergen, Jonas Lies Vei, 5020 Bergen, Norway

*corresponding author: ruth.brenk@uib.no

Manual druggability assignment for selected binding sites

Linezolid binding site in 50S ribosomal subunit

Linezolid is an FDA approved antibiotic targeting the 50S ribosomal subunit (Figure 1).[1] Based on its QED score of 0.89, it is highly drug-like. Its modest affinity of 20 µM translates to a LE of 0.27 kcal·mol^{-1}·heavy atom^{-1}. Linezolid is deeply buried in the pocket and forms mainly hydrophobic contacts in addition to a hydrogen bond to the ribose backbone of G2540 (Figure 4A). DrugPred_RNA predicted this pocket to be druggable. According to the individual SHAP values of the descriptor, the druggability was driven by the hydrophobicity of the pocket (*psa_r* = 0.32, *fr_hpb_atoms* = 0.77, *hsa* = 710 Å^2) and its shape (*PMI3* = 1,17 x 10^5, *fr_buried_sl_atoms* = 0.41) albeit the exposed surface area of the superligand being in a range that was more favourable for less druggable pockets (*exp_sl_sa* = 294 Å^2). Based on the binding mode of linezolid and the fact that linezolid is a drug-like ligand the prediction that this binding pocket is druggable appears to be valid, despite the ligand not binding as potently as expected for a drug.

FMN riboswitch binding site

The FMN riboswitch has been validated as a target for the antibiotic compound ribocil, a drug-like small molecule (QED score = 0.71, Figure 1).[2] The affinity for ribocil (K_D = 13 nM) is driven by hydrogen bonding with the base of A99 and the ribose group of A48 as well as stacking interactions with A85, A49 and, G62 (Figure 4B).[3] The binding site was rather deep (*fr_buried_sl_atoms* = 0.35) and characterized by a low relative polar surface area (*psa_r* = 0.33), a large the fraction of hydrophobic atoms (*fr_hpb_atoms* = 0.74), a rather large size of the hydrophobic contact surface area (*hsa* = 730 Å^2) and a large third principal moment of inertia (*PMI3* = 1.06 x 10^5). These values drove the site to be predicted as druggable despite its spherocity index lying in the less druggable range (*SpherocityIndex* = 0.41). The prediction agrees with the site binding drug-like ligands like ribocil with high ligand efficiency (LE = 0.41 kcal·mol^{-1}·heavy atom^{-1}).

TAR RNA binding site

A known ligand for the HIV-1 trans activating region (TAR) RNA is the drug acetylpromazine (QED = 0.85, Figure S5). Developed for a different target, the compound binds only with moderate affinity and efficiency to TAR RNA (K_D = 270 µM, LE = 0.22 kcal·mol^{-1}·heavy atom^{-1}).[4] In the structure of the complex, the ligand forms stacking interactions with U25 and U40 (Figure 4C). DrugPred_RNA predicted the ligand binding site to be druggable. As with the examples above, the classification was driven by a large fraction of hydrophobic atoms (*fr_hpb_atoms* = 0.78), the depth of the pocket (*fr_buried_sl_atoms* = 0.41), the high ratio of the superligand atoms to binding site atoms (*sl_bs_r* = 1.4) and the large third moment of inertia (*PMI3* = 4.65 x 10^4). These properties overcame the high solvent accessibility *(exp_sl_sa* = 506 Å^2), and the relative high polarity of the binding site (*psa_r* = 0.39). More potent ligands for HIV TAR RNA

are also known albeit structural information about their binding modes is lacking. Examples are a drug-like screening hit (QED = 0.72) and furimidazoline (QED = 0.72) which have affinities of 230 nM and 1 µM, resp. translating to LEs of 0.33 and 0.31 kcal·mol^{-1}·heavy atom^{-1} (Figure S5).[5,6] Assuming that these ligands bind into the same pocket as acetylpromazine, the prediction that this pocket is druggable appears to be reasonable.

Guanine and lysine riboswitch binding sites

Ligands binding to the guanine and lysine riboswitch have been shown to act as antibiotics.[7,8] In both cases, the pockets are rather small and almost fully enclose the natural ligands (Figure 4D and E). Structure-activity relationships (SAR) are very tight and only small modifications of the ligands are possible without losing binding affinity. DrugPred_RNA predicted these pockets to be less druggable which agrees with the SAR data. The predictions of the pockets were driven by their low relative polar surface areas (*psa_r* = 0.16 and 0.39, resp.), their lack of a sufficiently large hydrophobic surface area (*hsa* =109 Å^2 and 98 Å^2, resp.), small third principal moments of inertia (PMI3 = 357 and 859, resp.), their shallowness (*fr_buried_sl_atoms* = 0.0 in both cases), and their small size (*no_bs_atoms* = 37 and 59, *no_sl_atoms* = 8, 13, resp.).

Splicing site

Splicing modifiers for the treatment of spinal muscular atrophy are currently in clinical trials.[9,10] In our data set, the ligand SMN-C5 was included (Figure 4F). This ligand is moderate drug-like (QED = 0.55) and has a binding affinity of 28 µM translating to a LE of 0.22 kcal·mol^{-1}·heavy atom^{-1} for its target RNA. In the NMR structure, the flat ligand is lying in a highly solvent exposed binding site. DrugPred_RNA predicted this binding site to be less druggable. The prediction was due to the pocket being polar (*psa_r* = 0.46, *has* = 120 Å^2), shallow (*fr_buried_sl_atoms* = 0.11), and having an undesirable shape (*sl_bs_r* = 0.75, *PMI3* = 8.38 x 10^3). The druggability prediction appears to be reasonable considering the binding mode of the ligand, but not the fact that splicing modifiers are currently in clinical trials. This discrepancy is probably caused by the compounds binding *in vivo* to a ribonucleoprotein-RNA complex with a still unknown structure.[11] Thus, the biological relevant pocket of this type of compounds was not included in our study.

Paromomycin binding site in 16S ribosomal RNA

One class of FDA-approved ribosome binding antibiotics are aminoglycosides. One example of an aminoglycoside is paromomycin which acts by binding to the 16S ribosomal RNA (Figure 4G). Its low QED score of 0.11 is in agreement with the poor bioavailability of this compound class and the fact that aminoglycosides get into the bacteria by active transport.[12] In the complex of paromomycin bound to the ribosome of *T. thermophilus*, the ligand forms several hydrogen bonds with surrounding binding site

residues and water molecules (not shown), with little hydrophobic interactions. The terminal sugar ring in this ligand is located outside of the superligand created by DrugPred_RNA, suggesting that this area is a less optimal for ligand binding. The SHAP values suggested that despite the depth of the pocket (*fr_buried_sl_atoms* = 0.4) and the fraction of hydrophobic atoms (*fr_hpb_atoms* = 0.73) being in a range beneficial for druggable sites, the large polar surface area (*psa_r* = 0.51), the solvent-exposure (*exp_sl_sa* = 354 $Å^2$) combined with a less ideal shape (*InertialShapeFactor* = 1.10×10^{-4}, *sl_bs_r* = 1.2) contributed to the pocket being predicted as less druggable. This prediction agrees with the nature of the known ligands.

Tables

Table S1. Descriptors to describe size, polarity, and shape of ligand binding sites together with mean SHAP values for descriptors included in the final model.

**Descriptor name**	**Description**	**Descriptor type**	**Mean absolute SHAP value**
csa	Sum of SASA of binding site atoms	size	NA
psa	Sum of SASA of polar binding site atoms	polarity	NA
psa_r	*psa* / *csa*	polarity	1.46
hsa	Sum of SASA of hydrophobic binding site atoms	polarity/size	0.30
ali_sa_r	Sum of SASA of aliphatic binding site atoms / *csa*	polarity	NA
exp_sl_sa	Sum of SASA of superligand atoms that are solvent exposed in the superligand-receptor complex	shape	0.224
no_sl_atoms	Number of superligand atoms	size	0.202
no_bs_atoms	Number of binding site atoms	size	0.167
fr_buried_sl_atoms	Number of atoms buried inside the superligand / number of superligand surface atoms	shape	0.345
fr_hpb_atoms	Number of hydrophobic binding site atoms / no_bs_atoms	polarity	0.629
sl_bs_r	no_sl_atoms / no_bs_atoms	shape	0.193
vol	Volume of superligand	size	NA
sa_vol_r	Surface area of superligand / *vol*	shape	0.0907
PMI1	First principal moment of inertia	shape/size	NA
PMI2	Second principal moment of inertia	shape/size	NA
PMI3	Third principal moment of inertia	shape/size	0.277
NPR1	PM1 /PM3	shape	NA
NPR2	PM2 / PM3	shape	NA
Asphericity	$0.5\,\frac{(PM3-PM1)^2+(PM3-PM1)^2+(PM2-PM1)^2}{PM1^2+PM2^2+PM3^2}$	shape	NA

Eccentricity	$\frac{\sqrt{PM3^2 - PM1^2}}{PM3^2}$	shape	NA
SpherocityIndex	$\frac{3 \times PM1}{(PM1 + PM2 + PM3)}$	shape	0.0825
RadiusOfGyration	$\sqrt{\frac{2\pi^{\frac{PM3 \times PM2 \times PM1}{3}}}{MW}}$	shape	NA
InertialShapeFactor	$\frac{PM2}{PM1 \times PM3}$	shape	0.0849

Table S2. RNA families based on binding site sequence similarity. For each family, a head with PDB ID and three letter code of the small molecule bound to the pocket are listed. The total number of family members and the consensus score are also given. The druggability column contains the prediction that the majority of the members in each family obtained (RS = riboswitch).

Family	Head	Description	Members	Consensus score	Druggability
**1**	2gis_SAM	SAM-I RS	23	100.0	druggable
**2**	1j7t_PAR	Ribosomal binding site	17	41.2	less druggable
**3**	1y26_ADE	Adenine/Guanine RS	11	100.0	less druggable
**4**	1o9m_42B	Ribosomal binding site/ HIV-1 Kissing loops	18	88.9	less druggable
**5**	2yie_FMN	FMN RS	16	100.0	druggable
**6**	3ds7_GNG	Purine/ deoxyguanosine RS	8	50.0	less druggable
**7**	4qk8_2BA	c-di-AMP RS	10	45.5	less druggable
**8**	3irw_C2E	c-di-GMP RS	8	75.0	druggable
**9**	2ho7_G6P	glmS ribozyme	7	100.0	less druggable
**10**	2cky_TPP	TPP RS	16	12.5	Less druggable
**11**	1i9v_NMY	tRNA / Corn aptamer	4	100.0	less druggable
**12**	3d0u_LYS	Lysine RS	3	100.0	less druggable
**13**	4ts0_38E	Spinach aptamer	5	60.0	less druggable
**14**	6qiq_J48	CAG repeats	3	100.0	druggable
**15**	3e5c_SAM	SAM-III RS	3	100.0	druggable
**16**	4znp_AMZ	pfI/ZTP/ZMP RS	3	33.3	less druggable
**17**	1byj_GE1	Ribosomal sites	5	100.0	less druggable
**18**	3owi_GLY	Glycine RS	3	100.0	less druggable
**19**	1aju_ARG	HIV-2 Trans-Activating Region	3	100.0	less druggable
**20**	3suh_FFO	THF RS	3	100.0	less druggable
**21**	2ktz_ISH	HCV IRES	3	33.3	less druggable
**22**	6e8s_EKJ	Mango aptamer	2	0.0	-
**23**	6dmc_G4P	ppGpp RS	2	100.0	druggable
**24**	6c63_EKJ	Mango-II aptamer	2	100.0	druggable
**25**	5ny8_AGU	Guanidine-III RS	2	100.0	less druggable

**26**	5eao_CVC	Hammerhead ribozyme	2	100.0	druggable
**27**	2oe5_AM2	Human ribosomal site	2	100.0	less druggable
**28**	5ddp_GLN	Glutamine riboswitch	2	100.0	less druggable
**29**	2o43_ERN	Ribosomal site	2	100.0	druggable
**30**	4yaz_4BW	cGAMP RS	2	100.0	druggable
**31**	4qlm_2BA	ydaO RS	2	100.0	druggable
**32**	1nta_SRY	Streptomycin-binding aptamer	2	100.0	less druggable
**33**	5bjo_747	Corn aptamer	2	33.3	druggable
**34**	4l81_SAM	SAM-I/IV RS	2	100.0	druggable
**35**	4k32_GET	-	2	100.0	less druggable
**36**	4jf2_PRF	PreQ1-II RS	2	100.0	less druggable
**37**	5ux3_8OS	RNA hairpin	2	100.0	less druggable
**38**	3td1_GET	Protozoal cytoplasmic Ribosomal site	2	100.0	less druggable
**39**	6dlq_PRP	PRPP RS	2	100.0	druggable
**40**	3sd3_FFO	THF RS	3	100.0	less druggable
**41**	6e1t_HLV	PreQ1-I RS	2	0.0	-
**42**	3npn_SAH	SAH RS	2	100.0	druggable
**43**	1f1t_ROS	Malachite green aptamer	2	100.0	druggable
**44**	3gca_PQ0	PreQ1-I/PreQ0 RS	2	100.0	less druggable
**45**	3fu2_PRF	PreQ1-I RS	2	100.0	less druggable
**46**	1eht_TEP	Theophylline-binding RNA	2	100.0	druggable

Table S3. RNA families based on overall sequence similarity. For each family, a head with PDB ID and three letter code of the small molecule bound to the pocket are listed. The total number of family members and the consensus score are also given. The druggability column contains the prediction that the majority of the members in each family obtained. (RS = riboswitch)

Family	Head	Description	Members	Consensus score	Druggability
**1**	1j7t_PAR	Ribosomal binding site	21	33.3	less druggable
**2**	3dig_SLZ	Lysine RS	11	100.0	Less druggable
**3**	2eew_HPA	Guanine RS	11	100.0	Less druggable
**4**	3f4e_FMN	FMN RS	8	100.0	Druggable
**5**	3sd3_FFO	THF RS	8	100.0	Less druggable
**6**	6c8d_DGP	RNA-dGMP complex	5	100.0	Less druggable
**7**	3f2q_FMN	FMN RS	6	100.0	Less druggable
**8**	5nep_MGX	Guanidine RS	6	100.0	Less druggable
**9**	2hoj_TPP	TPP RS	7	42.86	Less druggable
**10**	1njn_SPS	Sparsomycin	6	100.0	Druggable
**11**	2gis_SAM	SAM RS	7	100.0	Druggable
**12**	2b57_6AP	Guanine RS	5	100.0	Less druggable
**13**	3owi_GLY	Glycine RS	5	100.0	Less druggable
**14**	4b5r_SAM	SAM RS	9	100.0	Druggable
**15**	2fcx_XXX	HIV-1 DIS	4	100.0	Less druggable
**16**	4tzx_ADE	Adenine RS	4	100.0	Less druggable
**17**	2cky_TPP	TPP RS	4	50.0	Druggable
**18**	6dlq_PRP	PRPP RS	4	50.0	Druggable
**19**	2ho7_G6P	glmS RZ	7	100.0	Less druggable
**20**	3ski_GNG	2-deoxyguanosine RS	4	100.0	Less druggable
**21**	3skl_GNG	2-deoxyguanosine RS	4	50.0	Less druggable
**22**	3irw_C2E	c-di-GMP RS	6	66.7	Druggable
**23**	3d0u_LYS	Lysine RS	4	100.0	Less druggable
**24**	6n5k_2BA	YdaO RS	3	33.3	Druggable
**25**	3e5c_SAM	SAM RS	3	100.0	Druggable
**26**	4y1i_GTP	Mn RS	3	100.0	Less druggable

**27**	4ts0_38E	Sprinach aptamer	4	50.0	Less druggable
**28**	4fel_HPA	Guanine RS	4	100.0	Less druggable
**29**	6e1s_HLV	PreQ1 RS	3	33.3	Druggable
**30**	5ddp_GLN	L-glutamine RS	2	100.0	Less druggable
**31**	4yaz_4BW	cGAMP RS	3	33.3	Druggable
**32**	3la5_5AZ	Mc6 RNA RS	3	100.0	Less druggable
**33**	6e8u_HZD	Mango RS	2	100.0	Druggable
**34**	6e1t_HLV	PreQ1 RS	2	0.0	-
**35**	6dmc_G4P	ppGpp RS	2	100.0	Druggable
**36**	3gca_PQ0	PreQ RS	2	100.0	Less duggable
**37**	1uud_P14	HIV-1 TAR	2	100.0	Less druggable
**38**	3fwo_MT9	Ribosomal subunit	2	100.0	Druggable
**39**	2gdi_TPP	TPP RS	2	100.0	Less druggable
**40**	1nta_SRY	Streptomycin	2	100.0	Less druggable
**41**	6c63_EKJ	Mango aptamer	2	100.0	Druggable
**42**	5eao_CVC	Hammerhead RZ	2	100.0	Druggable
**43**	4xw7_AMZ	ZMP RS	2	100.0	Less druggable
**44**	4qk8_2BA	c-di-AMP RS	2	100.0	Druggable
**45**	3bnq_PAR	Paromomycin	2	100.0	Less druggable
**46**	2yie_FMN	FMN RS	2	100.0	Druggable
**47**	3suh_FFO	THF RS	2	100.0	Less druggable
**48**	2o3v_N33	Ribosomal decoding site	2	100.0	Less druggable
**49**	6e81_TFX	Corn aptamer	2	100.0	Less druggable
**50**	2mxs_PAR	Neomycin RS	2	100.0	Druggable
**51**	2ktz_ISH	HCV IRES	2	100.0	Druggable
**52**	6qir_J48	CAG repeats	2	100.0	Druggable
**53**	6n5q_2BA	YdaO RS	2	100.0	Druggable
**54**	3mj3_SE4	IRES	2	100.0	Less druggable
**55**	6n5n_2BA	YdaO RS	2	0.0	-
**56**	6gzk_FH8	TMR aptamer	2	100.0	Druggable
**57**	1zz5_CNY	Neomycin	2	100.0	Less druggable

Table S4. Ribosomal binding site families based on binding site sequence similarity. For each family, a head with PDB ID and three letter code of the small molecule bound to the pocket together with its name are listed. The organism associated with the binding site, the total number of family members, and the consensus score are also given. The druggability column contains the prediction that the majority of the members in each family obtained.

Family	Head	Ligand	Organism	Members	Consensus	Druggability
**1**	1k8a_CAI	Carbomycin	*H. morismortui*	13	100.0	druggable
**2**	1jzx_CLY	Clindamycin	*D. radiodurans, T. thermophilus*	17	100.0	druggable
**3**	1fjg_PAR	Paromomycin	*T. thermophilus*	56	41.9	less druggable
**4**	1j5a_CTY	Clarithromycin	*D. radiodurans, T. thermophilus*	13	100	druggable
**5**	5jup_GDP	GDP	*S. cerevisiae*	9	77.8	less druggable
**6**	1ttt_GNP	GNP	*E. coli, T. aquaticus*	10	100.0	less druggable
**7**	4wfa_ZLD	Linezolid	*S. aureus*	7	100.0	druggable
**8**	6ole_MVM	PF846	*H. sapiens*	6	100.0	druggable
**9**	1j7t_PAR	Paromomycin	*16S rRNA Synthetic constructs*	9	11.1	druggable
**10**	1fjg_SRY	Spectinomycin	*T. thermophilus*	9	100.0	less druggable
**11**	4u3u_3HE	Cicloheximide	*S. cerevisiae*	6	100.0	druggable
**12**	1ibk_PAR	Paromomycin	*T. thermophilus*	30	72.4	less druggable
**13**	6gxm_GCP	GCP	*E. coli*	4	100.0	less druggable
**14**	4wpo_GDP	GDP	*T. thermophilus, E. coli*	6	100.0	less druggable
**15**	3jap_GCP	GCP	*K. lactis, S. cerevisiae*	4	100.0	less druggable
**16**	3id5_SAM	SAM/SAH	*S. solfataricus*	4	50.0	Less druggable
**17**	5zq0_SAH	SAH	*S. pneumoniae*	4	50.0	druggable
**18**	4v52_NMY	Neomycin	*E. coli, T. thermophilus*	4	0.00	-
**19**	6hiv_GTP	GTP	*T. brucei*	3	33.3	druggable
**20**	4u3m_ANM	Anisomycin	*S. cerevisiae*	8	100.0	druggable

**21**	1m90_SPS	Sparsomycin	*H. morismortui, S. cerevisiae*	6	66.7	druggable
**22**	5jup_SO1	Sordarin	*S. cerevisiae*	4	50.0	less druggable
**23**	6gaw_GSP	GSP	*S. scrufa, H. sapiens*	3	100.0	less druggable
**24**	5lzx_GCP	GCP	*H. sapiens*	3	100.0	less druggable
**25**	3jct_GTP	GTP	*S. cerevisiae*	4	100.0	less druggable
**26**	4wf1_NEG	Negamycin	*E. coli*	4	100.0	less druggable
**27**	1kqs_PPU	Puromycin	*H. morismortui*	3	100.0	druggable
**28**	6hiv_UTP	UTP	*T. brucei*	2	100.0	less druggable
**29**	4k32_GET	Geneticin	*Leishmania*	2	100.0	less druggable
**30**	4ji3_SRY	Streptomycin	*T. thermophilus*	2	100.0	less druggable
**31**	4io9_1F2	Carbomycin A derivative	*D. radiodurans*	2	100.0	druggable
**32**	1hnw_TAC	Tetracycline	*T. thermophilus*	2	100.0	less druggable
**33**	3wru_SJP	Neomycin analogue	-	2	100.0	less druggable
**34**	3td1_GET	Geneticin	-	2	100.0	less druggable
**35**	5lzb_GNP	GNP	*E. coli*	2	100.0	less druggable
**36**	5kcr_6UQ	Avilamycin	*E. coli*	2	100.0	less druggable
**37**	5jvg_6NO	Avilamycin	*D. radiodurans*	2	100.0	less druggable
**38**	5juu_SO1	Sodarin	*S. cerevisiae*	2	0.0	-
**39**	3oij_SAH	SAH	*S. cerevisiae*	2	100.0	less druggable
**40**	3jcj_GNP	GNP	*E. coli*	2	0.0	druggable
**41**	5aj4_GDP	GDP	*S. scrofa*	2	0.0	druggable
**42**	3jah_ADP	ADP	*O. corniculm, H. sapiens*	2	100.0	less druggable
**43**	3j7a_34G	Enetube	*P. falciparum*	2	100.0	less druggable
**44**	3cpw_ZLD	Linezolid	*H. morismortui*	2	100.0	druggable
**45**	4v9o_GCP	GCP	*T. thermophilus*	2	100.0	less druggable
**46**	2otj_13T	13-deoxytedanolide	*H. morismortui*	2	100.0	druggable
**47**	4v56_SCM	Spectinomycin	*E. coli*	2	100.0	less druggable
**48**	2g5k_AM2	Apramycin	*H. sapiens*	2	100.0	less druggable

**49**	4u56_BLS	Blasticidin S	*S. cerevisiae, T. Thermophilus*	2	100.0	less druggable
**50**	6rxt_GTP	GTP	*C. thermophilum*	2	100.0	less druggable
**51**	4u4y_PCY	Pactamycin, amicoumacin	*S. cerevisae*	2	100.0	druggable
**52**	6sg9_SAH	SAH	*T. brucei*	2	100.0	druggable

Figures

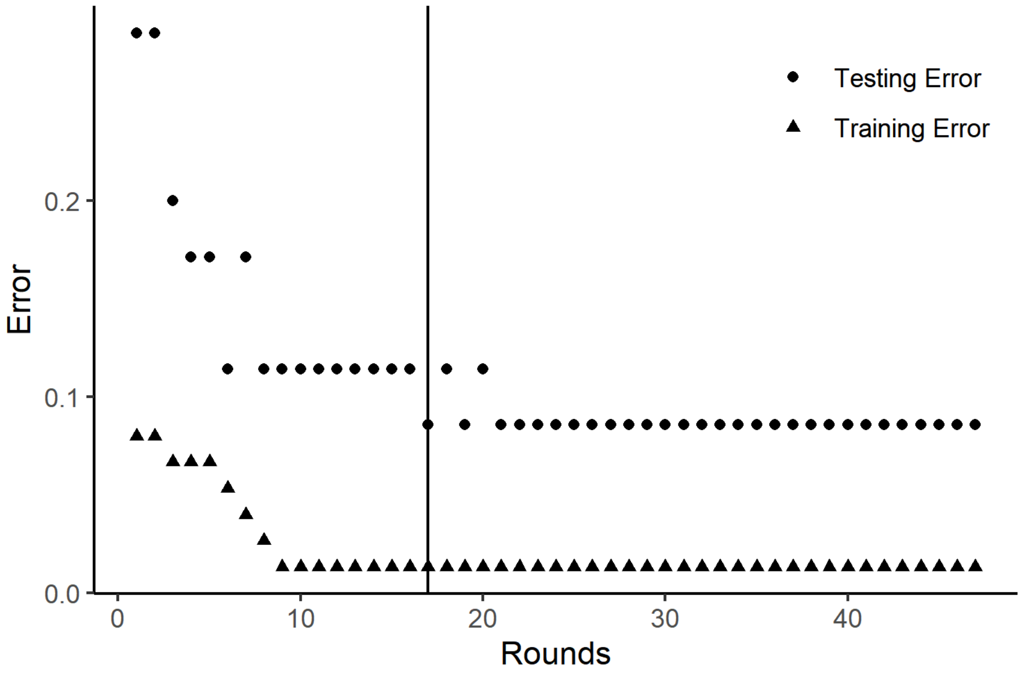

Figure S1. Training and test set accuracy error *vs*. rounds of XGboost iteration. The line denotes the earliest iteration (17) where the error on the test set has not improved in the following 20 iterations.

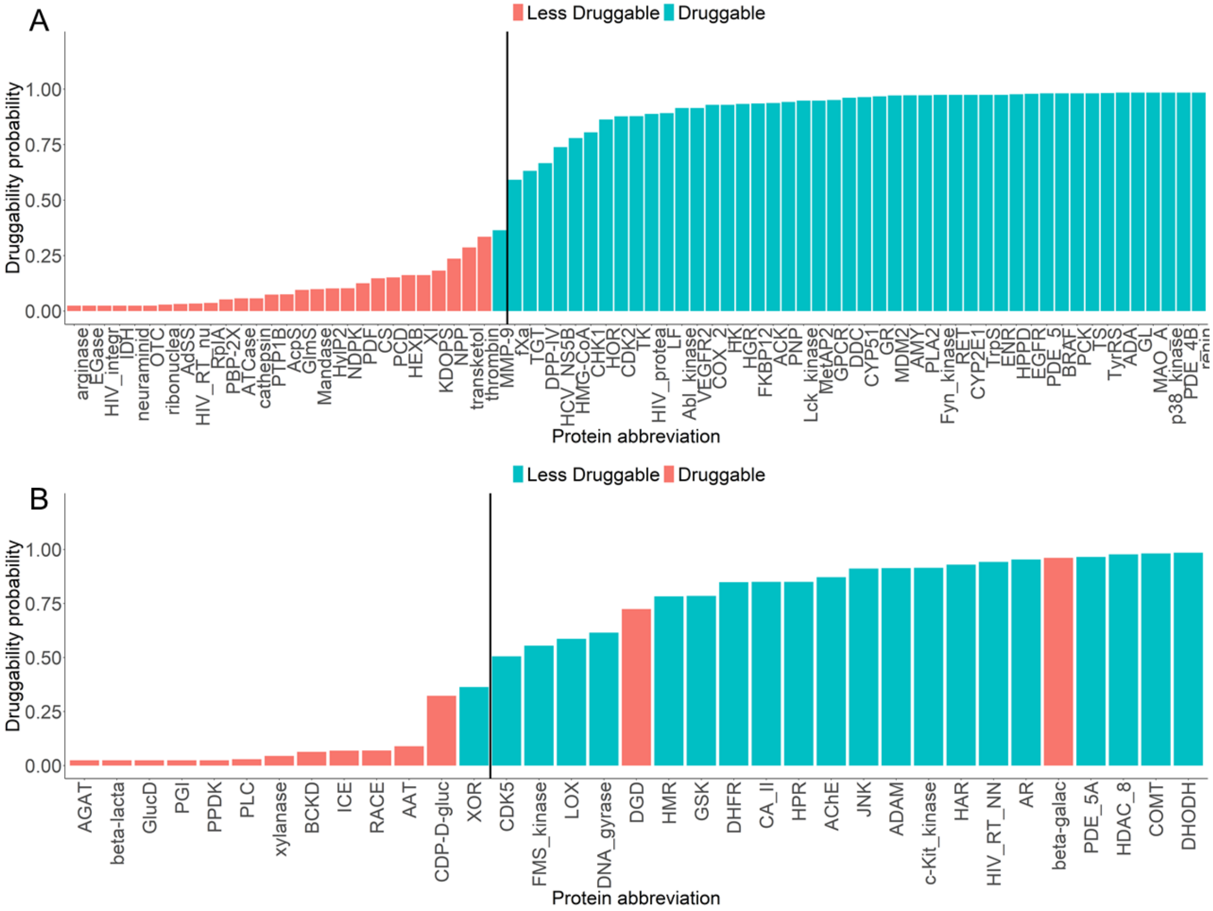

Figure S2. Druggability predictions with DrugPred_RNA for the NRDLD training (A) and test set (B). Cyan bars represent druggable and red bars less druggable binding sites. All pockets at the right side of the black line are classified as druggable while the pockets on the left side of the line are classified as less druggable. The full names of the proteins are listed in [13].

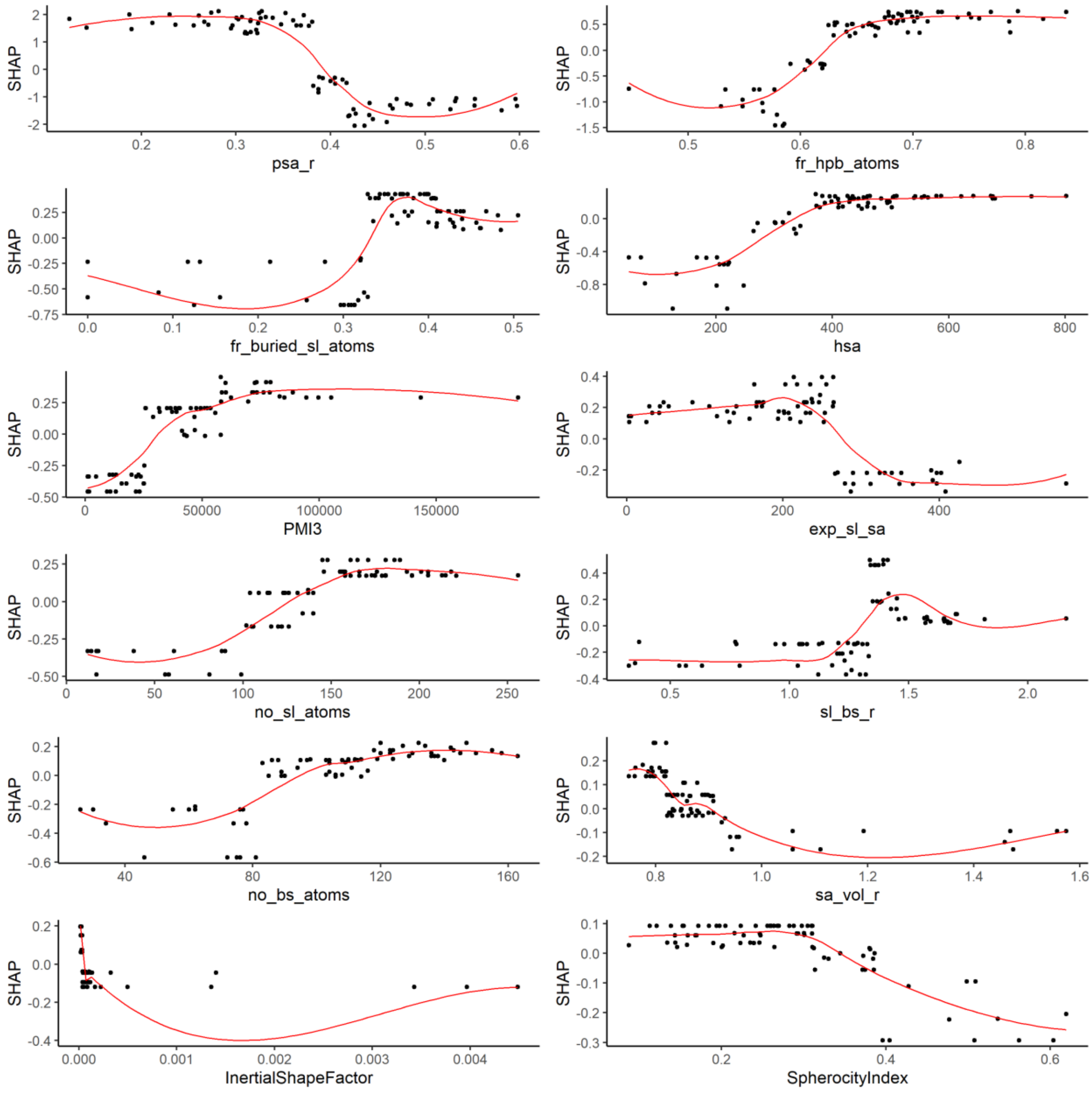

Figure S3. Individual SHAP values for each pocket in the training set for all descriptors in the final model plotted against the descriptor values. Locally estimated scatterplot smoothing (LOESS) curves are overlaid on the descriptor observations (black dots). The midpoint in each curve indicates the cut-off value from where the predictions change the direction.

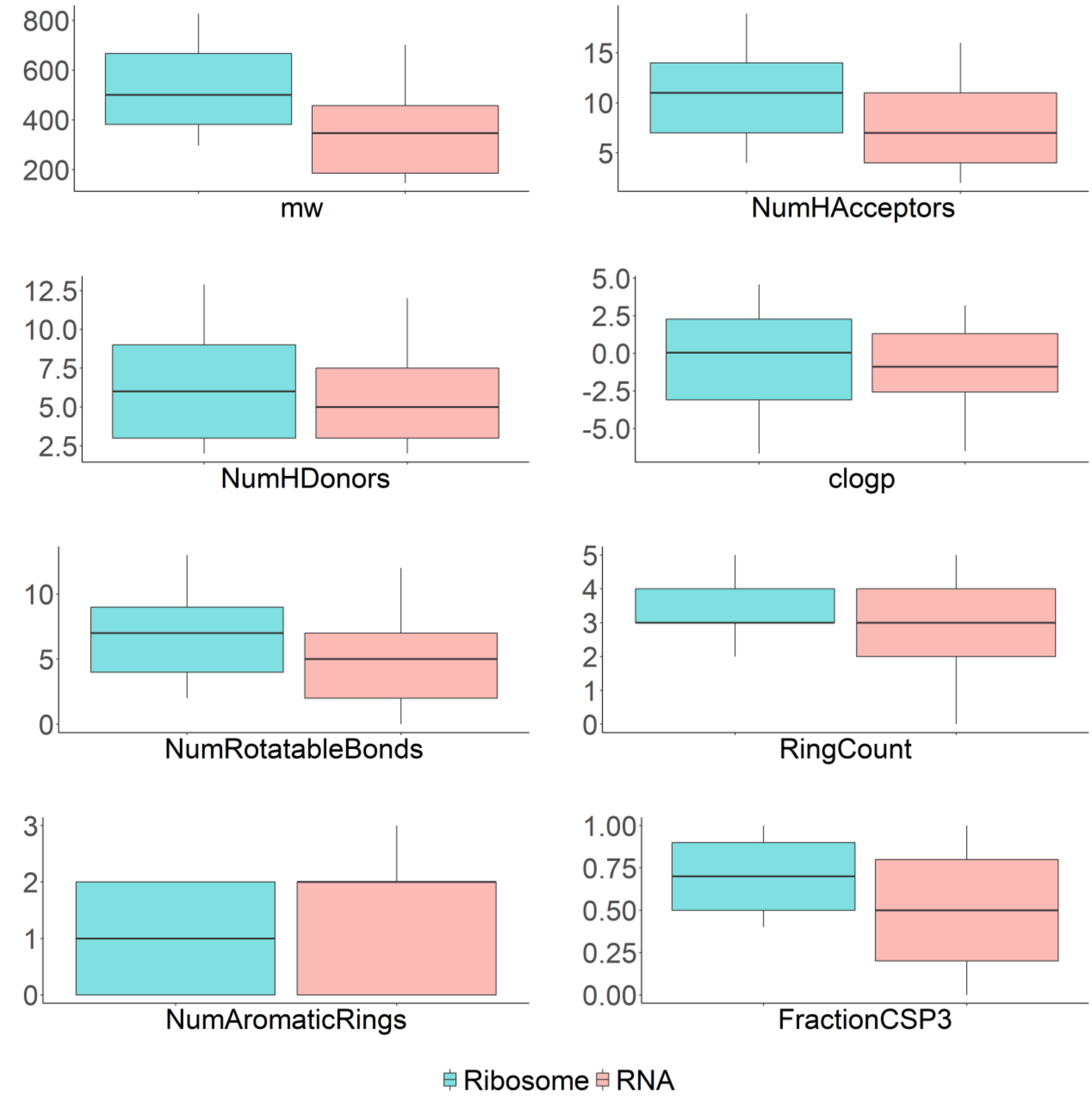

Figure S4. Boxplots showing the distribution of physico-chemical descriptors of ligands found in the ribosomal (green) and RNA-only (pink) data sets. The lower and upper hinges of the boxes represent the 25th and 75th percentiles of the data, and the whiskers extend to the bottom 10th and upper 90th percentile. The line inside the boxes marks the median value.

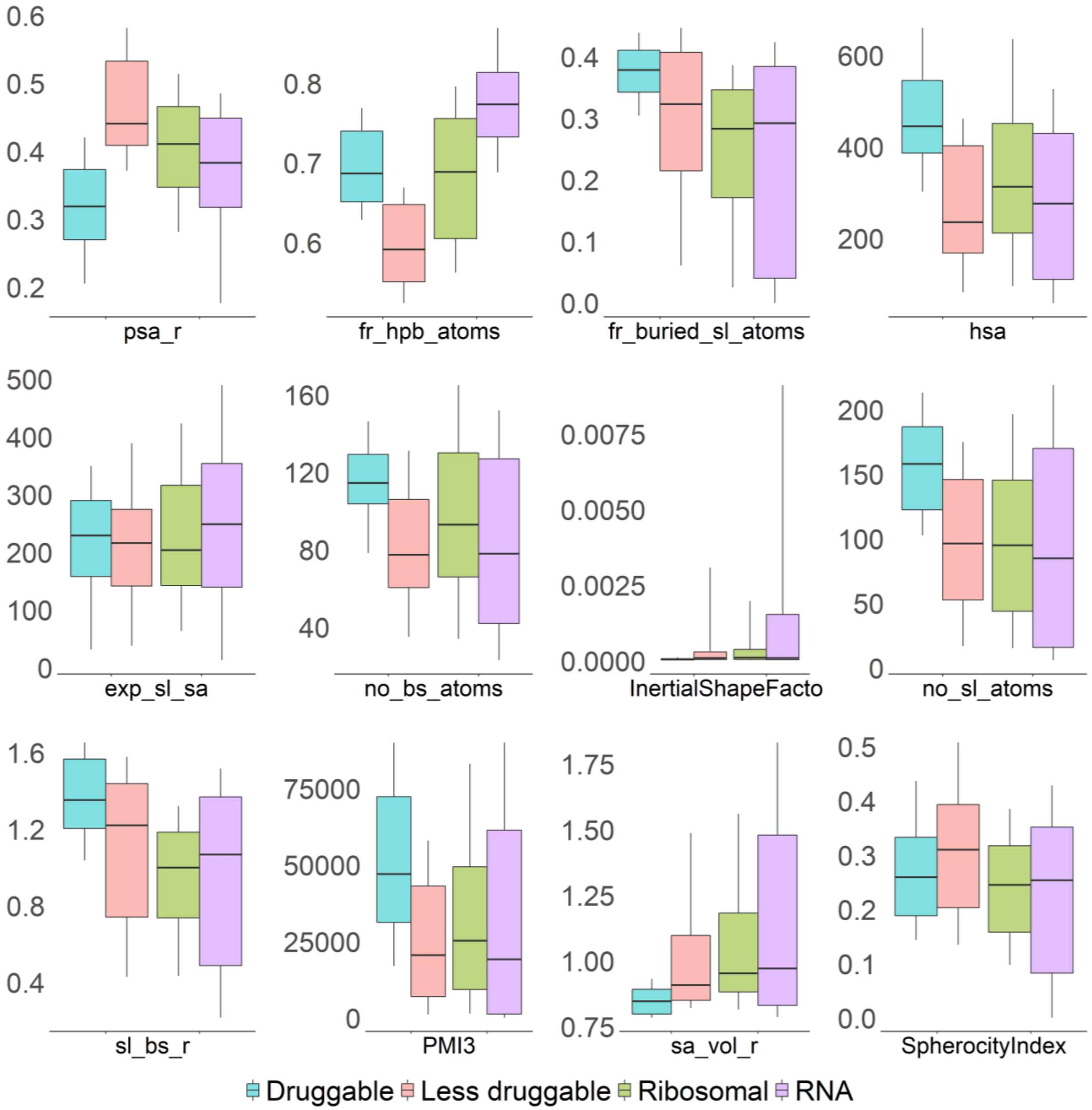

Figure S5: Boxplots showing the distribution of all descriptors in the DrugPred_RNA model for the protein binding sites in the NRDLD set and the RNA binding sites in the RNA sets. The lower and upper hinges of the boxes represent the 25th and 75th percentiles of the data, and the whiskers extend to the bottom 10th and upper 90th percentile. The line inside the boxes marks the median value.

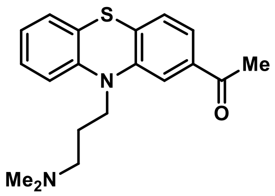

Acetylpromazine
QED = 0.85
K_D = 270 μM
LE = 0.22 kcal · mol $^{-1}$ · heavy atom^{-1}

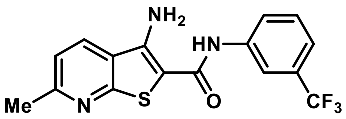

Screening hit
QED = 0.72
K_D = 230 nM
LE = 0.33 kcal · mol $^{-1}$ · heavy atom^{-1}

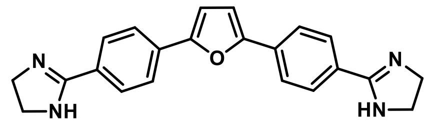

Furimidazoline
QED = 0.72
IC_{50} = 1 μM
LE = 0.33 kcal · mol $^{-1}$ · heavy atom^{-1}

Figure S6. Ligands of HIV-1 TAR RNA.

REFERENCES

(1) Ippolito, J. A.; Kanyo, Z. F.; Wang, D.; Franceschi, F. J.; Moore, P. B.; Steitz, T. A.; Duffy, E. M. Crystal Structure of the Oxazolidinone Antibiotic Linezolid Bound to the 50S Ribosomal Subunit. *J. Med. Chem.* **2008**, *51*, 3353–3356. https://doi.org/10.1021/jm800379d.

(2) Howe, J. A.; Wang, H.; Fischmann, T. O.; Balibar, C. J.; Xiao, L.; Galgoci, A. M.; Malinverni, J. C.; Mayhood, T.; Villafania, A.; Nahvi, A.; Murgolo, N.; Barbieri, C. M.; Mann, P. A.; Carr, D.; Xia, E.; Zuck, P.; Riley, D.; Painter, R. E.; Walker, S. S.; Sherborne, B.; de Jesus, R.; Pan, W.; Plotkin, M. A.; Wu, J.; Rindgen, D.; Cummings, J.; Garlisi, C. G.; Zhang, R.; Sheth, P. R.; Gill, C. J.; Tang, H.; Roemer, T. Selective Small-Molecule Inhibition of an RNA Structural Element. *Nature* **2015**, *526*, 672–677. https://doi.org/10.1038/nature15542.

(3) Howe, J. A.; Xiao, L.; Fischmann, T. O.; Wang, H.; Tang, H.; Villafania, A.; Zhang, R.; Barbieri, C. M.; Roemer, T. Atomic Resolution Mechanistic Studies of Ribocil: A Highly Selective Unnatural Ligand Mimic of the E. Coli FMN Riboswitch. *RNA Biol.* **2016**, *13*, 946–954. https://doi.org/10.1080/15476286.2016.1216304.

(4) Du Z; Lind KE; James Tl. Structure of TAR RNA Complexed with a Tat-TAR Interaction Nanomolar Inhibitor That Was Identified by Computational Screening. *Chem. Biol.* **2002**. https://doi.org/10.1016/s1074-5521(02)00151-5.

(5) Sztuba-Solinska, J.; Shenoy, S. R.; Gareiss, P.; Krumpe, L. R. H.; Le Grice, S. F. J.; O'Keefe, B. R.; Schneekloth, J. S. Identification of Biologically Active, HIV TAR RNA-Binding Small Molecules Using Small Molecule Microarrays. *J. Am. Chem. Soc.* **2014**, *136*, 8402–8410. https://doi.org/10.1021/ja502754f.

(6) Gelus, N.; Bailly, C.; Hamy, F.; Klimkait, T.; Wilson, W. D.; Boykin, D. W. Inhibition of HIV-1 Tat-TAR Interaction by Diphenylfuran Derivatives: Effects of the Terminal Basic Side Chains. *Bioorg. Med. Chem.* **1999**, *7*, 1089–1096. https://doi.org/10.1016/S0968-0896(99)00041-3.

(7) Blount, K. F.; Wang, J. X.; Lim, J.; Sudarsan, N.; Breaker, R. R. Antibacterial Lysine Analogs That Target Lysine Riboswitches. *Nat. Chem. Biol.* **2007**, *3*, 44–49. https://doi.org/10.1038/nchembio842.

(8) Yan, L.-H.; Le Roux, A.; Boyapelly, K.; Lamontagne, A.-M.; Archambault, M.-A.; Picard-Jean, F.; Lalonde-Seguin, D.; St-Pierre, E.; Najmanovich, R. J.; Fortier, L.-C.; Lafontaine, D.; Marsault, É. Purine Analogs Targeting the Guanine Riboswitch as Potential Antibiotics against Clostridioides Difficile. *Eur. J. Med. Chem.* **2018**, *143*, 755–768. https://doi.org/10.1016/j.ejmech.2017.11.079.

(9) Calder, A. N.; Androphy, E. J.; Hodgetts, K. J. Small Molecules in Development for the Treatment of Spinal Muscular Atrophy. *J. Med. Chem.* **2016**, *59*, 10067–10083. https://doi.org/10.1021/acs.jmedchem.6b00670.

(10) Ratni, H.; Karp, G. M.; Weetall, M.; Naryshkin, N. A.; Paushkin, S. V.; Chen, K. S.; McCarthy, K. D.; Qi, H.; Turpoff, A.; Woll, M. G.; Zhang, X.; Zhang, N.; Yang, T.; Dakka, A.; Vazirani, P.; Zhao, X.; Pinard, E.; Green, L.; David-Pierson, P.; Tuerck, D.; Poirier, A.; Muster, W.; Kirchner, S.; Mueller, L.; Gerlach, I.; Metzger, F. Specific Correction of Alternative Survival Motor Neuron 2 Splicing by Small Molecules: Discovery of a Potential Novel Medicine To Treat Spinal Muscular Atrophy. *J. Med. Chem.* **2016**, *59*, 6086–6100. https://doi.org/10.1021/acs.jmedchem.6b00459.

(11) Sivaramakrishnan, M.; McCarthy, K. D.; Campagne, S.; Huber, S.; Meier, S.; Augustin, A.; Heckel, T.; Meistermann, H.; Hug, M. N.; Birrer, P.; Moursy, A.; Khawaja, S.; Schmucki, R.; Berntenis, N.; Giroud, N.; Golling, S.; Tzouros, M.; Banfai, B.; Duran-Pacheco, G.; Lamerz, J.; Hsiu Liu, Y.; Luebbers, T.; Ratni, H.; Ebeling, M.; Cléry, A.; Paushkin, S.; Krainer, A. R.; Allain, F. H.-T.; Metzger, F. Binding to SMN2 Pre-MRNA-Protein Complex Elicits Specificity for Small Molecule Splicing Modifiers. *Nat. Commun.* **2017**, *8*, 1476. https://doi.org/10.1038/s41467-017-01559-4.

(12) Krause, K. M.; Serio, A. W.; Kane, T. R.; Connolly, L. E. Aminoglycosides: An Overview. *Cold Spring Harb. Perspect. Med.* **2016**, *6*. https://doi.org/10.1101/cshperspect.a027029.

(13) Krasowski, A.; Muthas, D.; Sarkar, A.; Schmitt, S.; Brenk, R. DrugPred: A Structure-Based Approach to Predict Protein Druggability Developed Using an Extensive Nonredundant Data Set. *J. Chem. Inf. Model.* **2011**, *51*, 2829–2842. https://doi.org/10.1021/ci200266d.
